# The *Pseudomonas Syringae* Effector AvrPtoB Associates With and Ubiquitinates *Arabidopsis* Exocyst Subunit EXO70B1

**DOI:** 10.3389/fpls.2019.01027

**Published:** 2019-08-29

**Authors:** Wei Wang, Na Liu, Chenyang Gao, Lu Rui, Dingzhong Tang

**Affiliations:** ^1^State Key Laboratory of Ecological Control of Fujian-Taiwan Crop Pests, Key Laboratory of Ministry of Education for Genetics, Breeding and Multiple Utilization of Crops, Plant Immunity Center, Fujian Agriculture and Forestry University, Fuzhou, China; ^2^Institute of Genetics and Developmental Biology, Chinese Academy of Sciences, Beijing, China; ^3^College of Life Sciences, University of Chinese Academy of Sciences, Beijing, China

**Keywords:** *Arabidopsis*, plant immunity, EXO70B1, AvrPtoB, TIR-NBS2, exocyst complex, NLR protein

## Abstract

Many bacterial pathogens secret effectors into host cells to disable host defenses and thus promote infection. The exocyst complex functions in the transport and secretion of defense molecules, and loss of function of the EXO70B1 subunit leads to autoimmunity by activation of a truncated Toll/interleukin-1 receptor–nucleotide-binding sequence protein (TIR-NBS2; herein referred to as TN2). Here, we show that EXO70B1 is required for pathogen-associated molecular pattern-triggered immune responses in *Arabidopsis thaliana*. The effector AvrPtoB, an E3 ligase from *Pseudomonas syringae* pv. *tomato* (*Pto*) strain DC3000, associates with EXO70B1. AvrPtoB ubiquitinates EXO70B1 and mediates EXO70B1 degradation *via* the host’s 26S proteasome in a manner requiring E3 ligase activity. AvrPtoB enhances *Pto* DC3000 virulence by overcoming EXO70B1-mediated resistance. Moreover, overexpression of AvrPtoB in *Arabidopsis* leads to autoimmunity, which is partially dependent on TN2. Expression of TN2 in tobacco (*Nicotiana tabacum* and *Nicotiana benthamiana*) triggers strong and rapid cell death, which is suppressed by co-expression with EXO70B1 but reoccurs when co-expressed with AvrPtoB. Taken together, our data highlight that AvrPtoB targets the *Arabidopsis thaliana* EXO70 protein family member EXO70B1 to manipulate the defense molecule secretion machinery or immunity.

## Introduction

Plants have evolved a multilayered immune system to cope with pathogens. For the initial immune response, conserved pathogen-derived molecules, termed pathogen-associated molecular patterns (PAMPs), are detected at the plant cell surface by the plasma membrane (PM)-bound receptor-like kinases (RLKs) or receptor-like proteins (RLPs), termed pattern recognition receptors (PRRs) (Wu and Zhou, 2013; Tang et al., 2017). Well-studied PRRs include the *Arabidopsis* leucine-rich repeat receptor kinases FLAGELLIN SENSING 2 (FLS2) and EF-TU RECEPTOR (EFR), which recognize bacterial flagellin and EF-Tu, respectively (Zipfel et al., 2004; Zipfel et al., 2006). After perception of PAMPs by the PRRs, a series of defense responses are activated, such as a burst of reactive oxygen species (ROS), an increase of Ca^2+^ concentration, callose deposition, mitogen-activated protein kinase (MAPK) activation, and pathogenesis-related gene expression, termed PAMP-triggered immunity (PTI) (Jones and Dangl, 2006; Tang et al., 2017). The BRASSINOSTEROID RECEPTOR-ASSOCIATED KINASE 1 (BAK1) interacts with FLS2 or EFR and functions as a co-receptor in PTI (Chinchilla et al., 2007; Sun et al., 2013).

To aid in infection, pathogens suppress PTI by secreting virulence effectors into the intercellular space or the cytoplasm of host cells and cause effector-triggered susceptibility (ETS) (Jones and Dangl, 2006). In the second layer of innate immunity, termed effector-triggered immunity (ETI), pathogen effectors are directly or indirectly recognized by the host mainly *via* intracellular nucleotide-binding (NB) leucine-rich repeat (LRR) domain receptors (NLRs); recognition of effectors by NLR proteins leads to robust immunity that terminates pathogen growth (Cui et al., 2015; Duxbury et al., 2016).

The gram-negative plant bacterial pathogen *Pseudomonas syringae* pv. *tomato* (*Pto*) strain DC3000 causes bacterial speck disease in tomato (*Solanum lycopersicum*) and *Arabidopsis thaliana* and injects about 30 effectors *via* the type III secretion system into host cells to suppress plant innate immunity (Nomura et al., 2006; Velasquez et al., 2017). AvrPto and AvrPtoB are two well-analyzed, sequence-distinct effectors from *Pto* DC3000. In tomato, both AvrPto and AvrPtoB interact with the tomato protein Pto, a serine/threonine protein kinase, and elicit Prf-mediated programmed cell death (PCD) and immunity (Kim et al., 2002; Xiao et al., 2007). AvrPtoB also targets a host protein kinase, Fen, to disrupt plant immunity (Rosebrock et al., 2007). In *Arabidopsis*, AvrPto and AvrPtoB suppress basal defense and intercept multiple PAMPs-mediated signaling upstream of MAPKKK (de Torres et al., 2006; He et al., 2006). AvrPto and AvrPtoB also can induce endogenous proteolytic activity that degrades RESISTANCE TO PSEUDOMONAS SYRINGAE PV MACULICOLA 1 (RPM1)-INTERACTING PROTEIN 4 (RIN4) in the presence of Pto and Prf (Luo et al., 2009). In *Arabidopsis* and tomato, AvrPto suppresses cell wall-based defenses and AvrPtoB promotes the virulence of *Pto* DC3000 by suppressing PCD (Abramovitch et al., 2003; Abramovitch et al., 2006; Xiang et al., 2008). AvrPto and AvrPtoB target multiple RLKs involved in the perception of PAMPs in *Arabidopsis*. For instance, AvrPto and AvrPtoB target BAK1 to interfere with the bacterial-induced formation of the FLS2-BAK1 receptor–signaling complex (Shan et al., 2008; Cheng et al., 2011). AvrPto targets BOTRYTIS-INDUCED KINASE 1 (BIK1), a downstream component of the FLS2 pathway, to prevent the phosphorylation of BIK1 (Xiang et al., 2011). AvrPtoB contains an N-terminal region at 1–307 amino acids (aa), which is sufficient to elicit Pto/Prf-mediated PCD (Xiao et al., 2007), and a C-terminal U-box type E3 ubiquitin ligase domain. AvrPtoB targets and degrades FLS2, EFR, and the LysM domain receptor kinase CHITIN ELICITOR RECEPTOR KINASE 1 (CERK1) to block the initiation of PTI signaling in *Arabidopsis* (Gohre et al., 2008; Gimenez-Ibanez et al., 2009). AvrPtoB ubiquitinates and degrades NON-EXPRESSER OF PR GENES 1 (NPR1) to block salicylic acid-dependent transcriptional reprogramming (Chen et al., 2017).

Targeted protein transport and coordinated membrane dynamics play an important role in PTI (Gu et al., 2017). These trafficking events are mediated by a large group of regulatory proteins, such as the exocyst complex, which may function in the transport of defense molecules and contributes to their secretion (Robatzek, 2007; Nathalie and Bouhidel, 2014). EXO70 is one of the eight subunits of the exocyst complex. The *Arabidopsis* genome encodes 23 EXO70 protein family members, some of which are involved in plant immunity (Cvrckova and Zarsky, 2013). For instance, the *exo70B1* loss-of-function mutant displays enhanced resistance to the powdery mildew pathogen *Golovinomyces cichoracearum*, the bacterial pathogen *Pto* DC3000, and the oomycete pathogen *Hyaloperonospora arabidopsidis* Noco2 (Zhao et al., 2015). EXO70B1 is also implicated in autophagy-related transport to the vacuole, and loss of function of EXO70B1 causes reduced numbers of internalized autophagosomes inside the vacuole (Kulich et al., 2013), along with ectopic hypersensitive responses (Kulich et al., 2013; Stegmann et al., 2013; Zhao et al., 2015). In addition, EXO70B1 positively regulates light- and drought-induced stomatal movement (Hong et al., 2016; Seo et al., 2016), and EXO70B1 is co-localized with EXO84B in broad bean (*Vicia faba*) guard cells (Hong et al., 2016). EXO70B2 and EXO70B1 show the highest sequence similarity, and both have been reported to be necessary for PTI and resistance to pathogens (Stegmann et al., 2012; Stegmann et al., 2013). Moreover, RIN4 recruits EXO70B1 to the plant cell PM (Sabol et al., 2017). The plant U-box-type ubiquitin ligase 22 (PUB22) ubiquitinates and degrades EXO70B2, which contributes to PAMP-triggered responses (Stegmann et al., 2012).

The activated immune responses in the *exo70B1* mutant require the atypical NLR protein Toll/interleukin-1 receptor–nucleotide-binding sequence protein (TIR-NBS2; herein referred to as TN2) and CALCIUM-DEPENDENT PROTEIN KINASE 5 (CPK5). Overexpression of CPK5 also leads to TN2-dependent autoimmune responses. Moreover, TN2 directly interacts with EXO70B1, and the expression level of *TN2* is up-regulated in mature *exo70B1-3* mutants (Zhao et al., 2015; Liu et al., 2017). TN2 belongs to the TIR-NBS (TN) family, which has 21 members in *Arabidopsis* ecotype Col-0 (Nandety et al., 2013; Kato et al., 2014). However, how TN2 contributes to *exo70B1*-activated resistance is still unclear. We proposed that EXO70B1 is a guardee of TN2 or the TN2-related complex and NLR-mediated immunity is activated when EXO70B1 is degraded (Zhao et al., 2015; Liu et al., 2017). To date, only two effectors are reported to target the components of the exocyst complex. In potato (*Solanum tuberosum*), *Phytophthora infestans* manipulates plant immunity by targeting the exocyst component Sec5 with the RXLR effector AVR1 (Du et al., 2015). In rice (*Oryza sativa*), the effector Avr-Pii from the rice blast fungus *Magnaporthe oryzae* interacts with OsEXO70F2 and OsEXO70F3, and the rice NLR protein Pii recognizes Avr-Pii and triggers OsEXO70F3-dependent immunity (Fujisaki et al., 2015).

Here, we show that EXO70B1 contributes to plant immunity triggered by multiple PAMPs and AvrPtoB interacts with EXO70B1 *via* its N-terminal domain. AvrPtoB ubiquitinates and mediates the degradation of EXO70B1. Overexpression of AvrPtoB in *Arabidopsis* leads to autoimmune phenotypes, which are partially dependent on TN2. AvrPtoB contributes to the virulence of *Pto* DC3000 by overcoming EXO70B1-mediated resistance. AvrPtoB can rescue TN2- or TN2-TIR-triggered cell death in tobacco, which is suppressed by EXO70B1. Taken together, these findings indicate that EXO70B1 functions positively in PTI signaling and is a substrate of AvrPtoB.

## Materials and Methods

### Plant Materials and Growth Conditions

The *Arabidopsis thaliana tn2-10* (SALK_204239C) mutant was obtained from the *Arabidopsis* Biological Resource Center. The *exo70B1-3* mutant and the *exo70B1-3* transgenic line containing a construct expressing EXO70B1-GFP under the control of the native *EXO70B1* promoter were described previously (Zhao et al., 2015). Double mutants were obtained by genetic crosses and identified by polymerase chain reaction (PCR). *Arabidopsis* plants were grown in a growth room at 20°C to 22°C and ∼60% relative humidity with a 16/8-h day/night photoperiod for seed setting and a 9/15-h day/night photoperiod for phenotyping, with a light intensity of 7,000 to 8,000 lux. *Nicotiana tabacum* and *Nicotiana benthamiana* plants were grown under the same short-day conditions as *Arabidopsis* (Wu et al., 2015).

### Vector Construction and Plant Transformation

The *AvrPtoB* sequence was amplified by PCR from *Pto* DC3000 genomic DNA and inserted into the pEASY vector using a pEASY Simple blunt Clonase Kit (TransGen Biotech). The *AvrPtoB^F479A^* mutant form was created using site-directed mutagenesis as described previously (Zhao et al., 2019). The *AvrPtoB* sequence was amplified with *Kpn*I and *Sal*I restriction sites and cloned into pSuper1300 with a 6 × Myc tag at the C-terminal to generate the* 35S:AvrPtoB-Myc* construct (Zhang et al., 2017). The *Agrobacterium tumefaciens* strain GV3101 carrying the construct was used to transform *Arabidopsis* plants by floral-dip transformation (Clough and Bent, 1998). The stably transformed plants carrying a single copy of the insert were identified in the T_3_ generation, and two independent stable transgenic lines AvrPtoB-Myc #2 and AvrPtoB-Myc #7 were chosen for further analysis.

### ROS Assays

ROS assays were performed as described previously (Zhang et al., 2010). Leaf strips of 4-week-old plants were treated with 100 nM of flg22, 100 nM of elf18, or 0.1 mg/ml of chitin. Luminescence was detected with the GloMax 96 microplate luminometer (Promega).

### Callose Deposition

Leaves from 4-week-old *Arabidopsis* plants were infiltrated with H_2_O, 20 μM of flg22, 20 μM of elf18, or 2 mg/ml of chitin in 10 mM of MgCl_2_ and removed 12 h after infiltration. Callose staining, image acquisition, and processing were carried out as described (Zhang et al., 2007).

### Y2H Assay

The Matchmaker GAL4 Two-Hybrid System 3 (Clontech) was used for yeast two-hybrid (Y2H) assays. Bacterial effector sequences were amplified from *Pto* DC3000 genomic DNA and were cloned into the vector pGBKT-7. The full-length *EXO70B1*, *EXO70B2*, and *EXO70A1* cDNA was cloned into pGADT-7. Different pairs of constructs were cotransformed into AH109, and the cells were grown on SD-Trp/-Leu medium. Three clones from SD-Trp/-Leu plates were transferred to SD-Trp/-Leu/-His/-Ade plates. Single clones were incubated in SD-Trp/-Leu liquid medium, and 10 μl of suspensions (diluted to OD_600_ = 0.5) of the overnight cultures was dropped onto different plates to test the interactions of the proteins.

### RT-qPCR

Total RNA extraction and quantitative reverse transcription PCR (RT-qPCR) were performed as described previously (Shi et al., 2013).

### Pull-Down Assay

The pull-down assay was performed as described previously (Yu et al., 2016). The *AvrPtoB* and *AvrPtoB_1–410_* sequences were amplified from *AvrPtoB-pEASY*, and the *AvrPtoB^F479A^* sequence was amplified from *AvrPtoB^F479A^-pEASY* with *Eco*RI and *Not*I restriction sites and were cloned into the prokaryotic expression vector pGEX-4T-1. The positive clones were selected by sequencing. The GST-AvrPtoB, GST-AvrPtoB^F479A^, and GST-AvrPtoB_1–410_ fusion proteins were expressed in *Escherichia coli* as described previously (Xie et al., 1999). The *EXO70B1* sequence was amplified from the *EXO70B1-AD* plasmid with *Kpn*I and *Sal*I restriction sites and cloned into pSuper1300 with a GFP tag at the C-terminal. EXO70B1-GFP was transiently expressed in *N. benthamiana* leaves by *Agrobacterium tumefaciens*-mediated infiltration at OD_600_ = 1.5, and samples were collected after 2 days. EXO70B1-GFP was extracted in native extraction buffer as described previously (Shi et al., 2013). Equimolar amounts of glutathione *S*-transferase (GST), GST-AvrPtoB, GST-AvrPtoB^F479A^, and GST-AvrPtoB_1–410_ were incubated with equal amounts of EXO70B1-GFP extracts at 4°C for 4 h. After incubation, the GST binding beads (Novagen) were added to incubate for another 2 h. Then, the mix was washed three times using GST washing buffer (40 mM of Na_2_HPO_4_, 8.5 mM of KH_2_PO_4_, 1.4 M of NaCl, 27 mM of KCl [pH 7.3], and 5 mM of dithiothreitol [DTT]). After the last centrifugation, the GST washing buffer was removed. Eighty microliters of GST washing buffer and 20 μl of 5 × SDS sample buffer were added, and the beads were boiled for 10 min. The proteins were separated by sodium dodecyl sulfate–polyacrylamide gel electrophoresis (SDS-PAGE) and then detected using anti-GST (Abmart) and anti-GFP (Abmart) antibodies.

### Co-IP Assay in *N. Benthamiana*

Coimmunoprecipitation (Co-IP) assays were performed as described previously, with minor modifications (Shi et al., 2013). Two days after infiltration, leaves were syringe infiltrated with or without 50 μM of MG132 for 3 h before freezing in liquid nitrogen. For anti-Myc immunoprecipitation, protein extracts were incubated with agarose-conjugated anti-Myc antibody (MBL) for 4 h at 4°C with gentle rotation. The agarose beads were washed and resuspended in 80 μl of PBS and 20 μl of 5 × SDS sample buffer and boiled for 10 min. Protein samples were detected with anti-GFP (Abmart) and anti-Myc (Abmart) immunoblot.

### Cell-Free and *In Vivo* Protein Degradation Assay

The *35S:AvrPtoB-Myc* construct or an empty vector control construct was transiently expressed in *N. benthamiana* leaves by *Agrobacterium tumefaciens*-mediated infiltration at OD_600_ = 1.0, and samples were collected after 2 days. Total leaf proteins were extracted in native extraction buffer. The *EXO70B1* sequence was amplified from the *EXO70B1-AD* plasmid with *Bam*HI and *Sal*I restriction sites and were cloned into the prokaryotic expression vector pMAL-c2g-1, and the MBP-EXO70B1 fusion protein was expressed in *E. coli*. Maltose-binding protein (MBP) and the MBP-EXO70B1 recombinant protein were incubated with a cell-free crude extract for 1.5 and 3 h in the presence or absence of 50 μM of MG132 (AG Scientific). The reactions were terminated by adding 5 × SDS sample buffer. The protein degradation patterns were analyzed by immunoblotting with anti-MBP (New England Biolabs) antibody, and AvrPtoB-Myc protein was detected by anti-Myc (Abmart) antibody.

For the *in vivo* protein degradation assay, the *EXO70B1* coding sequence was inserted into the PUC19 vector with a HA tag using *Sac*I and *Sal*I restriction sites. The *35S:EXO70B1-HA* and *35S:GFP* plasmids were cotransfected with the *35S:AvrPtoB-Myc* or *35S:AvrPtoB^F479A^-Myc* construct into protoplasts prepared from rosette leaves of wild-type *Arabidopsis* plants using the polyethylene glycol (PEG) method (Ryu et al., 2010). After incubation for 12 h, the protoplasts were treated with MG132 (50 μM) or dimethyl sulfoxide (DMSO) for 3 h. The total proteins were extracted in native extraction buffer, and then 5 × SDS sample buffer was added. The patterns of protein degradation were analyzed by immunoblot analysis with anti-HA (Abmart) and anti-GFP (Abmart) antibodies. AvrPtoB-Myc was detected by anti-Myc (Abmart) antibody.

### *In Vitro* Ubiquitination

*In vitro* ubiquitination was carried out as described (Gimenez-Ibanez et al., 2009; Stegmann et al., 2012). In brief, each reaction of 60 μl of final volume contained ubiquitination buffer (50 mM of Tris–HCl [pH 7.5], 5 mM of MgCl_2_, 2 mM of DTT, 4 mM of ATP, and 1 × protease inhibitor cocktail) and 1 μg of MBP-EXO70B1 or MBP-EXO70B2 in the presence or absence of 15 μg of ubiquitin, 50 ng of human E1 (UBE1), 200 ng of *Arabidopsis* E2 His-UBC8, and 500 ng of GST-AvrPtoB or GST-AvrPtoB^F479A^. The reactions were incubated at 30°C for 3 h and stopped by adding 5 × SDS sample buffer and boiling for 10 min. Samples were separated by an 8% SDS-PAGE gel followed by detection of ubiquitinated substrate by immunoblotting using anti-MBP (New England Biolabs) and anti-GST (Abmart) antibodies.

### Pathogen Infection Assays

Bacterial strains were grown overnight at 28°C in King’s B medium with appropriate antibiotics. Seedling infection assays were performed as described previously (Lu et al., 2010). In brief, seedlings were grown in 1/2 MS liquid medium for 10 days in a 12-well tissue culture plate. Bacteria were added at the concentration of OD_600_ = 0.2. Bacterial counting was performed 3 days after inoculation.

For powdery mildew infection, *Golovinomyces cichoracearum* strain UCSC1 was used to infect *Arabidopsis* plants at a high spore density (Adam and Somerville, 1996). The infected leaves were stained with Trypan Blue at 8 days postinfection to visualize hyphae and dead cells (Frye and Innes, 1998).

### Gene Expression

For gene expression analysis, *Arabidopsis* leaves of Col-0 plants were hand-infiltrated with *Pto* DC3000 or the *Pto ΔAvrPtoB* mutant at OD_600_ = 0.2 in 10 mM of MgCl_2_. Total RNA extraction and RT-qPCR were performed as described above.

### Hypersensitive Response Assay

*Agrobacterium* strain GV3101 was injected into 4-week-old *N. benthamiana* or *N. tabacum* leaves. The injected leaves were removed at 4 days postinoculation and photographed, then stained using Trypan Blue to examine cell death, and photographed.

### Oligonucleotide Sequences

The primers used in this study are listed in **Supplementary Table 1**.

## Results

### EXO70B1 Associates With AvrPtoB

**Figure 1:**
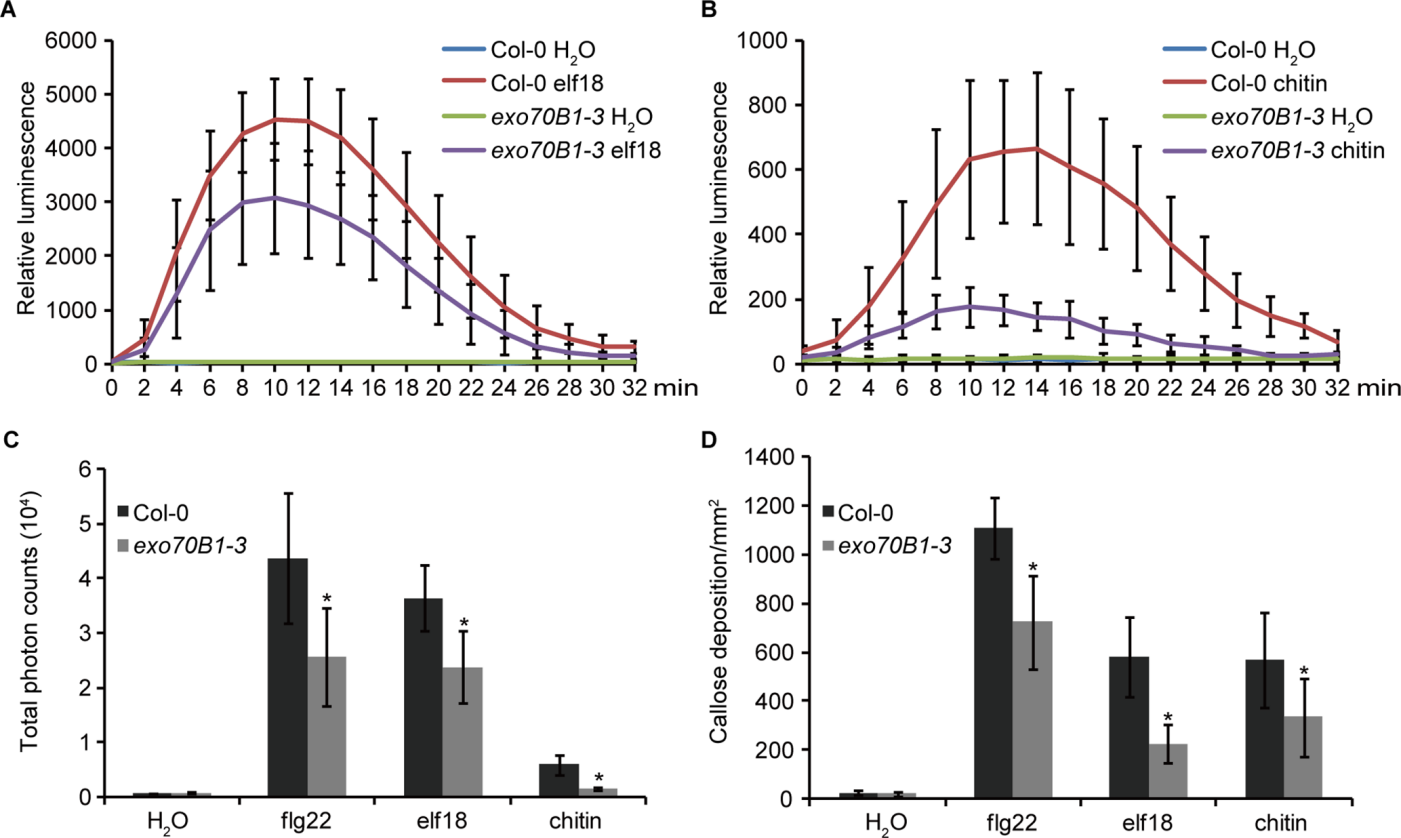
EXO70B1 is required for responses triggered by multiple PAMPs. **(A)** and **(B)** Luminescence-based assays of ROS production in leaves of four-week-old Col-0 and *exo70B1-3 Arabidopsis* plants following treatment with 100 nM of elf18 **(A)** or 0.1 mg/ml of chitin **(B)**. The means and standard deviations were determined using 12 independent biological samples. **(C)** Flg22-, elf18-, chitin-, and control (H_2_O)-induced ROS production over 30 min, presented as total photon counts in Col-0 and *exo70B1-3* mutants. Data represent the mean and standard deviation of eight independent biological samples. Asterisk indicates a statistically significant difference between Col-0 and *exo70B1-3* mutants (*P* < 0.05; Student’s *t*-test). **(D)** Flg22-, elf18-, chitin-, and control (H_2_O)-induced callose deposition in Col-0 and *exo70B1-3* mutants. Data represent the mean and standard deviation of 27 independent biological samples. Asterisk indicates a statistically significant difference between Col-0 and *exo70B1-3* mutants (*P* < 0.05; Student’s *t*-test). These experiments were repeated three times with similar results.

EXO70B1 is required for bacterial flagellin peptide 22 (flg22)-triggered immunity in plants (Stegmann et al., 2012). To further investigate the role of EXO70B1 in PTI, we examined elf18- and chitin-induced ROS production and callose deposition in the *exo70B1-3* mutant. When treated with elf18 or chitin, the* exo70B1-3* mutant showed reduced production of ROS than did the wild type Col-0 (**Figures 1A–C**). Similarly, flg22-, elf18-, and chitin-induced callose deposition was also significantly reduced in the *exo70B1-3* mutant (**Figure 1D**), indicating that EXO70B1 functions positively in multiple PAMPs-triggered responses in *Arabidopsis*.

Because of the positive role of EXO70B1 in PTI, we hypothesized that EXO70B1 may be targeted by pathogen effectors. To validate this hypothesis, we performed a Y2H screen to identify bacterial effectors that interact with EXO70B1. We identified several effectors from *Pto* DC3000 that interact with EXO70B1, including HopH1 (Wei et al., 2007), HopK1 (He et al., 2004), HopO1-1 (Guo et al., 2005), HopY1 (Guttman et al., 2002), AvrPto (Kim et al., 2002; He et al., 2006), and AvrPtoB^F479A^, the E3 ligase-deficient mutant of AvrPtoB (Gohre et al., 2008; Gimenez-Ibanez et al., 2009) (**Table 1**). According to our hypothesis, TN2 recognizes effectors by monitoring the status of EXO70B1, and the TN2-mediated resistance is activated when EXO70B1 disappeared (Zhao et al., 2015; Liu et al., 2017). Then, we focused on AvrPtoB as AvrPtoB is a well-characterized effector, which contains an E3 ligase domain in its C-terminal. EXO70B1 interacted with the E3 ligase-deficient mutant form of AvrPtoB only, indicating that EXO70B1 may be a substrate of wild-type AvrPtoB.

**Table 1:** EXO70B1 interacting effectors from *Pto* DC3000 identified by a Y2H screening.

Effectors	Accession number	Size (aa)	Characterization	Virulence
AvrPto	AAO57459	164	Unknown	Yes
AvrPtoB^F479A^	AY074795	553	E3 ligase deficient mutant	No
HopH1	AAO54130	218	Protease	Yes
HopK1	AAO53599	338	Unknown	No
HopO1-1	AF458392	283	ADP-Ribosyltransferase	Yes
HopY1	AF458403	287	Unknown	Unknown

**Figure 2:**
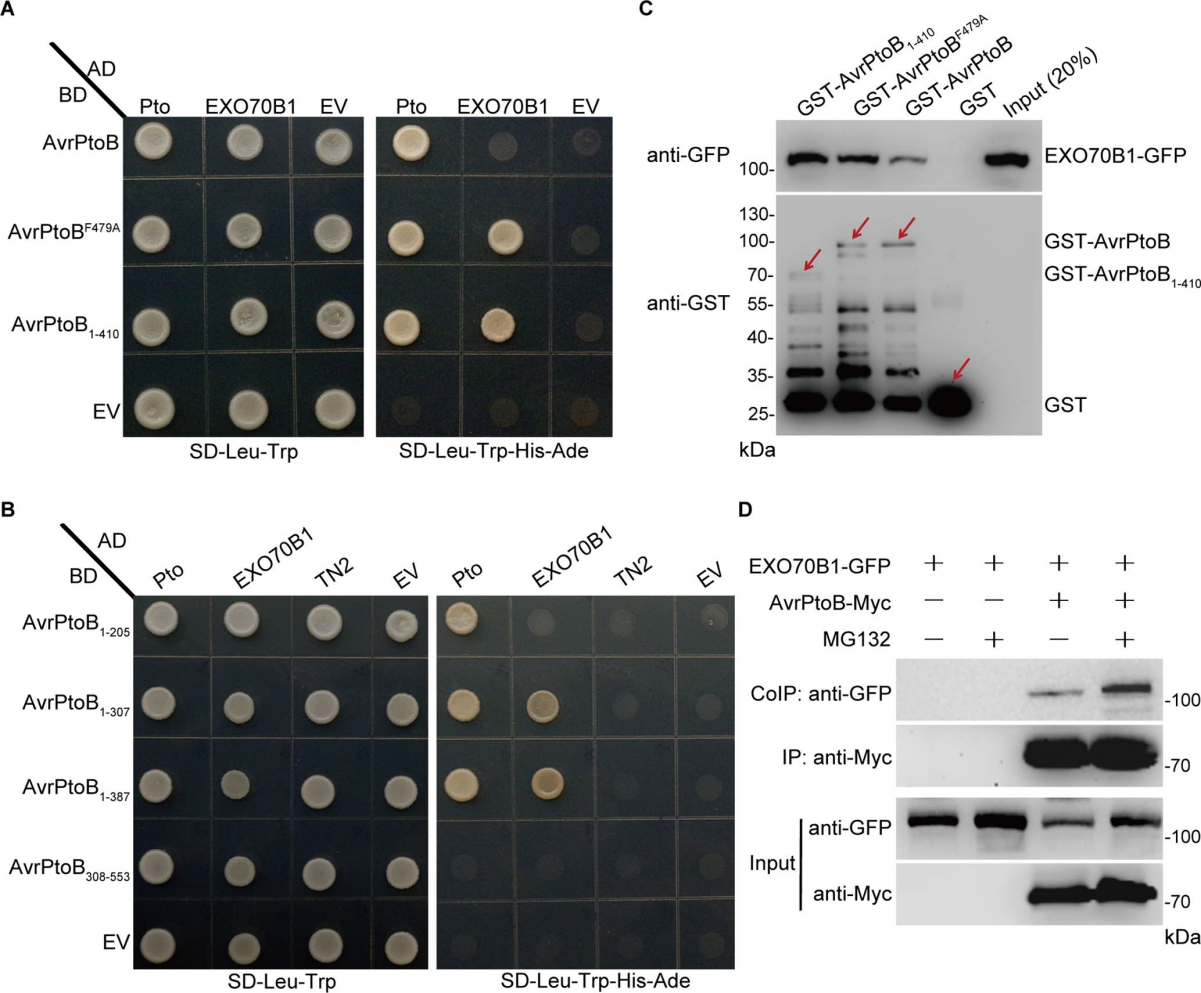
EXO70B1 associates with AvrPtoB. **(A)** EXO70B1 interacts with AvrPtoB in a Y2H assay. The coding sequences of AvrPtoB, AvrPtoB^F479A^, and AvrPtoB_1–410_ were fused to the Gal4 DNA binding domain (BD), and the coding sequence of EXO70B1 was fused to the Gal4 transactivation domain (AD). As a positive control, tomato Pto was also fused to the AD. Different pairs of constructs were cotransformed into AH109. A 10-μl suspension (OD_600_ = 0.5) of each cotransformant was dropped onto synthetic dropout (SD) medium lacking Leu and Trp and SD medium lacking Ade, His, Leu, and Trp. Photographs were taken after 5 days of incubation. **(B)** Amino acids 1–307 of AvrPtoB are necessary for the interaction with EXO70B1 in a Y2H assay. Different truncated fragments of AvrPtoB (1–205, 1–307, 1–387, and 308–553) were fused to the BD, and EXO70B1 and TN2 were fused to the AD. Different pairs of constructs were cotransformed into AH109. Yeast cells containing the indicated plasmids were spotted onto SD-Leu-Trp and SD-Ade-His-Leu-Trp. Photographs were taken after 5 days of incubation. **(C)** EXO70B1 interacts with AvrPtoB in a GST-pull-down assay. *E. coli*-expressed GST, GST-AvrPtoB, GST-AvrPtoB^F479A^, and GST-AvrPtoB_1–410_ were incubated with plant-expressed EXO70B1-GFP in pull-down assays. After incubation for 4 h at 4°C, the beads were washed three times, and 5 × SDS sample buffer was added. Then, 20 μl of the samples was utilized to detect the precipitated EXO70B1-GFP by anti-GFP antibody, and 10 μl of the samples was used to detect the levels of GST, GST-AvrPtoB, GST-AvrPtoB^F479A^, and GST-AvrPtoB_1–410_ by anti-GST antibody. Arrows indicate the positions of the corresponding proteins. **(D)** EXO70B1 interacts with AvrPtoB in a Co-IP assay. EXO70B1-GFP was co-expressed with AvrPtoB-Myc in *N. benthamiana* leaves. After 48 h, leaves were treated with or without 50 μM of MG132 for 3 h. Total protein was extracted and subjected to immunoprecipitation of AvrPtoB by anti-Myc antibody. Proteins were analyzed in an immunoblot using anti-GFP and anti-Myc antibodies. These experiments were repeated three times with similar results.

AvrPtoB contains two major domains and several specific domains in the N-terminus (**Supplementary Figure S1A**). AvrPtoB interacts with several target proteins through its N-terminal domains and degrades them *via* E3 ubiquitin ligase activity (Gohre et al., 2008; Gimenez-Ibanez et al., 2009; Chen et al., 2017). To test whether AvrPtoB interacts with EXO70B1 *via* its N-terminal domains, we introduced constructs encoding the N-terminal region lacking the E3 ligase domain of AvrPtoB (AvrPtoB_1–410_) as well as the E3-ligase-deficient mutant AvrPtoB^F479A^ into yeast (*Saccharomyces cerevisiae*) along with constructs encoding EXO70B1. Both forms of AvrPtoB interacted with EXO70B1 (**Figure 2A**), indicating that AvrPtoB maybe associated with EXO70B1 *via* one of the protein interacting domains in its N-terminal region. We did not observe that the full-length AvrPtoB interacts with EXO70B1 in Y2H assay, maybe because of the AvrPtoB E3 ligase activity, which may affect protein interaction in yeast.

Previously, we showed that EXO70B1 associates with TN2, and CPK5 phosphorylates EXO70B1 (Zhao et al., 2015; Liu et al., 2017). To examine whether AvrPtoB also interacts with TN2 or CPK5, we introduced constructs encoding AvrPtoB, AvrPtoB_1–410_, and AvrPtoB^F479A^ into yeast along with TN2 or CPK5, with Pto as a positive control. None of these constructs interacted with TN2 or CPK5 (**Supplementary Figure S1B**). Taken together, these data indicated that AvrPtoB interacts with EXO70B1, but not TN2 or CPK5, *via* its N-terminal region, and the E3 ligase domain of AvrPtoB is dispensable for the interaction.

To identify the specific domains of AvrPtoB that interact with EXO70B1 and to further confirm that there is no interaction between AvrPtoB and TN2, we generated a series of AvrPtoB truncations, including AvrPtoB_1–205_, AvrPtoB_1–307_, AvrPtoB_1–387_, and AvrPtoB_308–553_ (**Supplementary Figure S1A**). Two truncated proteins, AvrPtoB_1–307_ and AvrPtoB_1–387_, interacted with EXO70B1, and none of the deletion mutants interacted with TN2 (**Figure 2B**). As a positive control, Pto did interact with AvrPtoB_1–205_, which indicated that amino acid residues 1–307 of AvrPtoB are sufficient for the interaction between AvrPtoB and EXO70B1.

To test biochemically whether AvrPtoB interacts with EXO70B1, we performed GST-pull-down assays. The *35S:EXO70B1-green fluorescent protein* (*GFP*) vector was transiently expressed in *Nicotiana benthamiana* leaves by *Agrobacterium tumefaciens*-mediated infiltration. Total leaf proteins were extracted, and EXO70B1-GFP was detected by immunoblotting using anti-GFP antibody. Bacterially expressed GST, GST-AvrPtoB, GST-AvrPtoB^F479A^, and GST-AvrPtoB_1–410_ recombinant proteins were incubated with total proteins prepared from *N. benthamiana* leaves transiently expressing EXO70B1-GFP. GST protein was used as the negative control. GST-AvrPtoB, GST-AvrPtoB^F479A^, and GST-AvrPtoB_1–410_ could pull down EXO70B1-GFP, and the EXO70B1-GFP precipitated by GST-AvrPtoB^F479A^ and GST-AvrPtoB_1–410_ is increased possibly because of the degradation activity of wild-type AvrPtoB (**Figure 2C**).

To further test the interaction between AvrPtoB and EXO70B1 *in planta*, we performed Co-IP assays by transiently expressing EXO70B1-GFP and AvrPtoB-Myc in *N. benthamiana* leaves, using EXO70B1-GFP alone as a negative control. AvrPtoB was immunoprecipitated using anti-Myc antibody, and EXO70B1 was detected with anti-GFP antibody only in the precipitate from the leaves that co-expressed both EXO70B1-GFP and AvrPtoB-Myc, but not from the negative control leaves that only expressed EXO70B1-GFP. Interestingly, the protein level of EXO70B1 and the association of EXO70B1 and AvrPtoB were markedly increased upon treatment with MG132, an inhibitor of the proteasome complex (Bogyo et al., 1997) (**Figure 2D**). These results indicated that EXO70B1 and AvrPtoB form a protein complex in *N. benthamiana*, and AvrPtoB may mediate the degradation of EXO70B1.

### AvrPtoB Degrades EXO70B1 in a Proteasome-Dependent Manner

**Figure 3:**
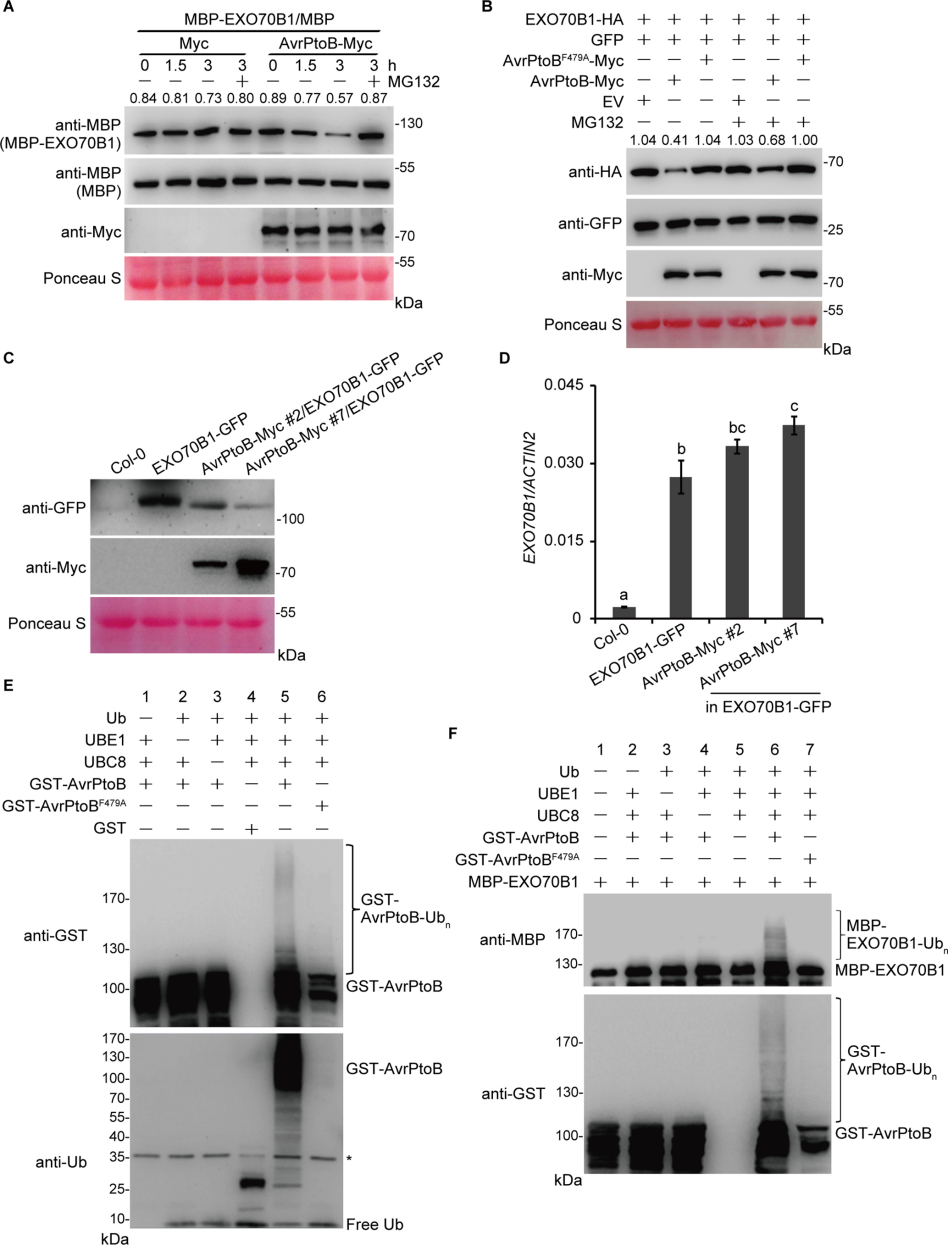
The degradation and ubiquitination of EXO70B1 by AvrPtoB. **(A)** Cell-free degradation assay of EXO70B1 by AvrPtoB. Bacterially expressed MBP or MBP-EXO70B1 was incubated with a cell-free crude extract prepared from *N. benthamiana* leaves transiently expressing AvrPtoB-Myc or Myc, in the presence or absence of 50 μM of MG132. The time-dependent changes of protein levels were monitored by immunoblotting with anti-MBP antibody. Numbers indicate the protein levels of MBP-EXO70B1 normalized to MBP using ImageJ software. The levels of Rubisco are shown as an equal loading control of cell-free extracts. **(B)** *In vivo* degradation assay of EXO70B1 by AvrPtoB in protoplasts. The *35S:EXO70B1-HA* and *35S:GFP* constructs were cotransfected with the *35S:Myc*, *35S:AvrPtoB-Myc*, or *35S:AvrPtoB^F479A^-Myc* constructs into protoplasts freshly isolated from wild-type *Arabidopsis* leaf tissues using PEG-mediated transformation. After overnight transfection, protoplasts were incubated with or without 50 μM of MG132 for 3 h. Total proteins were extracted and examined by immunoblot analysis with anti-HA, anti-GFP, and anti-Myc antibodies. The levels of GFP show the transfection efficiency of protoplasts. Numbers indicate the protein levels of EXO70B1-HA normalized to GFP using ImageJ software. The levels of Rubisco are shown as an equal loading control. **(C)** EXO70B1-GFP and AvrPtoB-Myc levels in the indicated genotypes were determined by immunoblotting using anti-GFP and anti-Myc antibodies. Ponceau S staining of Rubisco is shown as a protein loading control. **(D)** RT-qPCR analysis of *EXO70B1* expression. Total RNA was isolated from leaves of the 5-week-old *Arabidopsis* plants. The expression levels of *EXO70B1* were normalized to the reference gene *ACTIN2*. Data represent the mean and standard deviation of three biological replicates. Three technical replicates for each biological sample were used. The lowercase letters indicate statistically significant differences (*P* < 0.05; one-way ANOVA). **(E)** *In vitro* self-ubiquitination assay of AvrPtoB. Recombinant GST-AvrPtoB was incubated in ubiquitination buffer in the presence or absence of ubiquitin, E1 (UBE1), or E2 (UBC8) at 30°C for 2 h. Recombinant GST-AvrPtoB^F479A^ was used as a negative control. Reaction products were resolved using SDS-PAGE and subjected to immunoblot analysis with anti-GST and anti-Ub antibodies. Asterisk indicates the contaminating bands. **(F)** *In vitro* ubiquitination assay of EXO70B1 by AvrPtoB. Recombinant MBP-EXO70B1 was incubated in ubiquitination buffer in the presence or absence of ubiquitin, E1 (UBE1), E2 (UBC8), or E3 (AvrPtoB or AvrPtoB^F479A^). The reaction mixtures were subjected to immunoblot analysis with anti-MBP and anti-GST antibodies. Ubiquitinated MBP-EXO70B1 was detected by anti-MBP antibody. These experiments were repeated three times with similar results.

The interaction between EXO70B1 and AvrPtoB suggests that EXO70B1 may be a substrate of AvrPtoB and may be degraded by AvrPtoB. To investigate this possibility, we first conducted a cell-free degradation assay. The *35S:AvrPtoB-Myc* or an empty vector control construct was transiently expressed in *N. benthamiana* leaves. Total leaf proteins were extracted, and AvrPtoB-Myc was identified by immunoblotting using anti-Myc antibody. Bacterially expressed MBP or MBP-EXO70B1 recombinant proteins were incubated with total proteins prepared from *N. benthamiana* leaves transiently expressing AvrPtoB-Myc or Myc with or without MG132 and were subjected to immunoblot analysis with anti-MBP antibody at the indicated time. After 1.5 h of incubation, the level of MBP-EXO70B1 was slightly reduced in the AvrPtoB-Myc cell-free extracts. After 3 h of incubation, the level of MBP-EXO70B1 was substantially reduced in the AvrPtoB-Myc cell-free extracts. By contrast, MBP-EXO70B1 remained stable in the Myc cell-free extracts and in the presence of MG132 (**Figure 3A**), indicating that MBP-EXO70B1 is degraded in a proteasome-dependent manner in the presence of AvrPtoB-Myc. The MBP protein, as a negative control, remained unaffected in both extracts.

To further confirm that AvrPtoB mediates the degradation of EXO70B1, we next performed a protoplast transient expression assay. The EXO70B1-HA and GFP proteins were transiently co-expressed with AvrPtoB-Myc or AvrPtoB^F479A^-Myc in protoplasts prepared from *Arabidopsis* Col-0 leaves. After overnight transfection, protoplasts were incubated with or without MG132 for another 3 h. Total proteins were extracted and examined by immunoblotting analysis with anti-HA, anti-GFP, and anti-Myc antibodies. The level of EXO70B1-HA was decreased in the presence of AvrPtoB-Myc, but not AvrPtoB^F479A^-Myc (**Figure 3B**). The amount of EXO70B1-HA was increased in the presence of MG132 (**Figure 3B**), suggesting that the degradation of EXO70B1-HA by AvrPtoB-Myc *in vivo* in an E3-ligase-dependent manner is controlled by the 26S proteasome. The level of GFP, which was used to assess the efficiency of protoplast transfection, and Rubisco, which was shown as an equal protein loading control, remained unchanged regardless of the presence of MG132. Taken together, these data indicated that the degradation of EXO70B1 is regulated by AvrPtoB in a proteasome-dependent manner.

To further analyze whether AvrPtoB regulates the degradation of EXO70B1 in *Arabidopsis*, we introduced the *35S:AvrPtoB-Myc* clone into the *exo70B1-3* transgenic line, which contains a construct encoding functional EXO70B1-GFP in the *exo70B1-3* mutant background (Zhao et al., 2015). Several independent stable transgenic lines were obtained, and the *35S:AvrPtoB-Myc* #2 and #7 plants were chosen for further research. We extracted total proteins from 5-week-old Col-0, the *exo70B1-3* transgenic line, and the *35S:AvrPtoB-Myc* #2 and #7 plants and detected EXO70B1-GFP and AvrPtoB-Myc by immunoblotting analysis with anti-GFP and anti-Myc antibodies, respectively. The *35S:AvrPtoB-Myc* #2 and #7 transgenic plants expressed AvrPtoB-Myc of the correct size, and the amount of EXO70B1-GFP was lower in the *35S:AvrPtoB-Myc* transgenic plants compared with the controls (**Figure 3C**). We also tested the transcript levels of *EXO70B1* and found that they were slightly increased in the *35S:AvrPtoB-Myc* #2 and #7 plants (**Figure 3D**), indicating that AvrPtoB destabilizes EXO70B1 by post-translational regulation.

To investigate whether EXO70B1 is a substrate of AvrPtoB, we performed *in vitro* ubiquitination assays to analyze whether AvrPtoB can ubiquitinate EXO70B1. First, we used human E1 (UBE1) and *Arabidopsis* E2 (UBC8) to assess the *in vitro* E3 ligase activity of AvrPtoB. Recombinant full-length GST-AvrPtoB and GST-AvrPtoB^F479A^ were incubated with ubiquitination buffer in the presence or absence of ubiquitin (Ub), UBE1, and UBC8 at 30°C for 2 h. The reaction mixture was analyzed by immunoblotting using anti-Ub and anti-GST antibodies. We observed GST-AvrPtoB E3 ligase activity as evidenced by high-molecular-mass smear ladders detected by both anti-Ub and anti-GST antibodies (**Figure 3E**), indicating that GST-AvrPtoB could self-ubiquitinate in our experimental conditions. In contrast, the E3-ligase-deficient mutation of AvrPtoB, GST-AvrPtoB^F479A^, did not have E3 ligase activity (**Figure 3E**).

Next, a bacterially expressed MBP-EXO70B1 recombinant protein was used for *in vitro* ubiquitination assays in the presence of all the other components required for the ubiquitination reaction. After 3 h of incubation, multiple ubiquitinated forms of MBP-EXO70B1 appeared in the presence of Ub, UBE1, UBC8, and GST-AvrPtoB. However, coincubation of MBP-EXO70B1 with GST-AvrPtoB^F479A^ failed to produce any detectable high-molecular-mass smear ladders (**Figure 3F**), suggesting that the presence of an active E3 ligase domain is required for the ubiquitination of MBP-EXO70B1 by GST-AvrPtoB. Taken together, these data indicated that EXO70B1 is a substrate of AvrPtoB and is ubiquitinated by AvrPtoB.

Since EXO70B1 and EXO70B2 show the highest sequence similarity and both are required for PTI responses (Stegmann et al., 2012), we also analyzed whether AvrPtoB targets EXO70B2. With Pto as a positive control, we found that EXO70B2 also interacted with AvrPtoB (**Supplementary Figure S2A**). Next, with MBP-EXO70B1 as a positive control, no ubiquitination form of MBP-EXO70B2 was detected in the presence of all required ubiquitination components in our experimental condition (**Supplementary Figure S2B**). Taken together, these data indicated that EXO70B1, not EXO70B2, is a substrate of AvrPtoB.

### Overexpression of AvrPtoB Leads to Stunted Phenotypes in *Arabidopsis*

**Figure 4:**
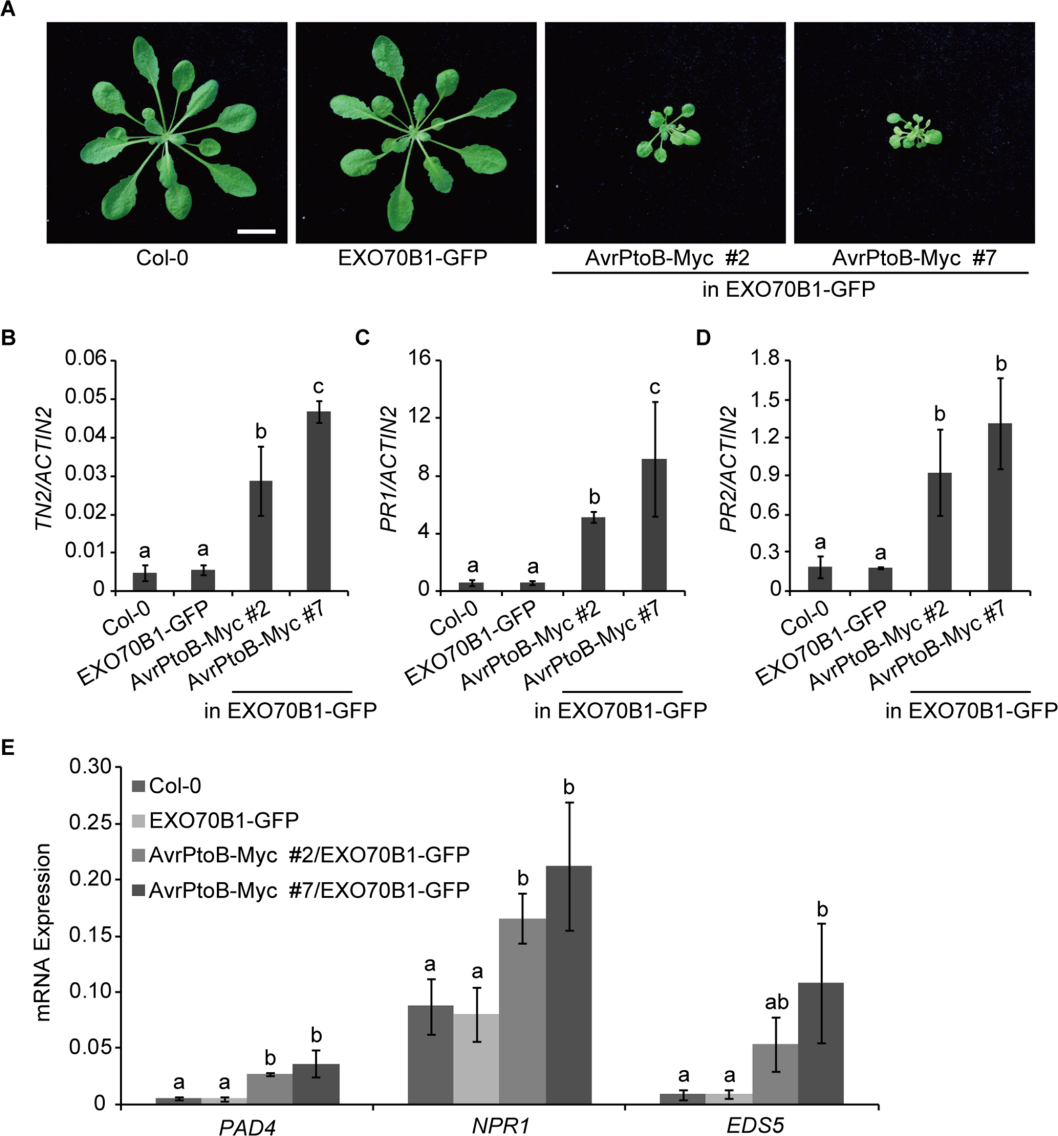
Overexpression of AvrPtoB leads to stunted phenotypes in *Arabidopsis*. **(A)** Five-week-old plants of Col-0, the *exo70B1-3* transgenic line (expressing EXO70B1-GFP in the *exo70B1-3* mutant background), and two independent *35S:AvrPtoB-Myc* transgenic *Arabidopsis* plants in the background of the *exo70B1-3* transgenic line were photographed under short-day conditions. Bar = 1.0 cm. **(B–E)** RT-qPCR analysis of *TN2* **(B)**, *PR1* **(C)**, *PR2* **(D)**, and immune-related genes **(E)** expression. Total RNA was isolated from leaves of the 5-week-old *Arabidopsis* plants indicated in **(A)**. The expression levels of indicated gene were normalized to the reference gene *ACTIN2*. Data represent the mean and standard deviation of three biological replicates. Three technical replicates for each biological sample were used. The lowercase letters indicate statistically significant differences (*P* < 0.05; one-way ANOVA). These experiments were repeated three times with similar results.

Further on, we analyzed the phenotypes of transgenic *Arabidopsis* plants that consistently express AvrPtoB. Compared with Col-0 and the *exo70B1-3* transgenic line, 5-week-old *35S:AvrPtoB-Myc* #2 and #7 transgenic plants displayed stunted phenotypes and growth defects (**Figure 4A**), which were similar to those in TN2 overexpressing plants (Li et al., 2009; Nandety et al., 2013). However, no obvious spontaneous cell death was observed in the *35S:AvrPtoB-Myc* plants.

Next, we analyzed the transcript levels of *TN2* in 5-week-old Col-0, the *exo70B1-3* transgenic line, and the *35S:AvrPtoB-Myc* #2 and #7 plants and found that the expression of *TN2* was significantly up-regulated in the *35S:AvrPtoB-Myc* #2 and #7 plants compared with the wild type and the *exo70B1-3* transgenic line (**Figure 4B**). In *exo70B1-3* mutant, the expression of *PR1* and *PR2* is constitutively up-regulated due to the activated TN2 (Zhao et al., 2015; Liu et al., 2017). Similarly, we found that the expression levels of *PR1* and *PR2* were also significantly up-regulated in the *35S:AvrPtoB-Myc* #2 and #7 plants (**Figures 4C, D**). We further examined the expression of other immune-related genes, including *PHYTOALEXIN DEFICIENT 4* (*PAD4*), *NPR1*, and *ENHANCED DISEASE SUSCEPTIBILITY 5* (*EDS5*). The transcript levels of these genes were significantly up-regulated in the *35S:AvrPtoB-Myc* #2 and #7 plants (**Figure 4E**). As shown in **Figure 3C**, AvrPtoB-Myc #7 accumulated much higher AvrPtoB-Myc protein levels and lower EXO70B1-GFP protein levels than did AvrPtoB-Myc #2, and the transcript levels of immune-related genes in AvrPtoB-Myc #7 were higher than those in AvrPtoB-Myc #2, which indicated that the stunted phenotypes and the up-regulation of immune-related genes in AvrPtoB-Myc plants are related to the low EXO70B1 protein levels. Taken together, these data indicated that overexpression of AvrPtoB leads to low accumulation of EXO70B1 and autoimmunity phenotypes, which may be caused by TN2-triggered plant immunity.

### The Autoimmunity Phenotypes in the AvrPtoB Overexpression Lines Are Partially Dependent on TN2

**Figure 5:**
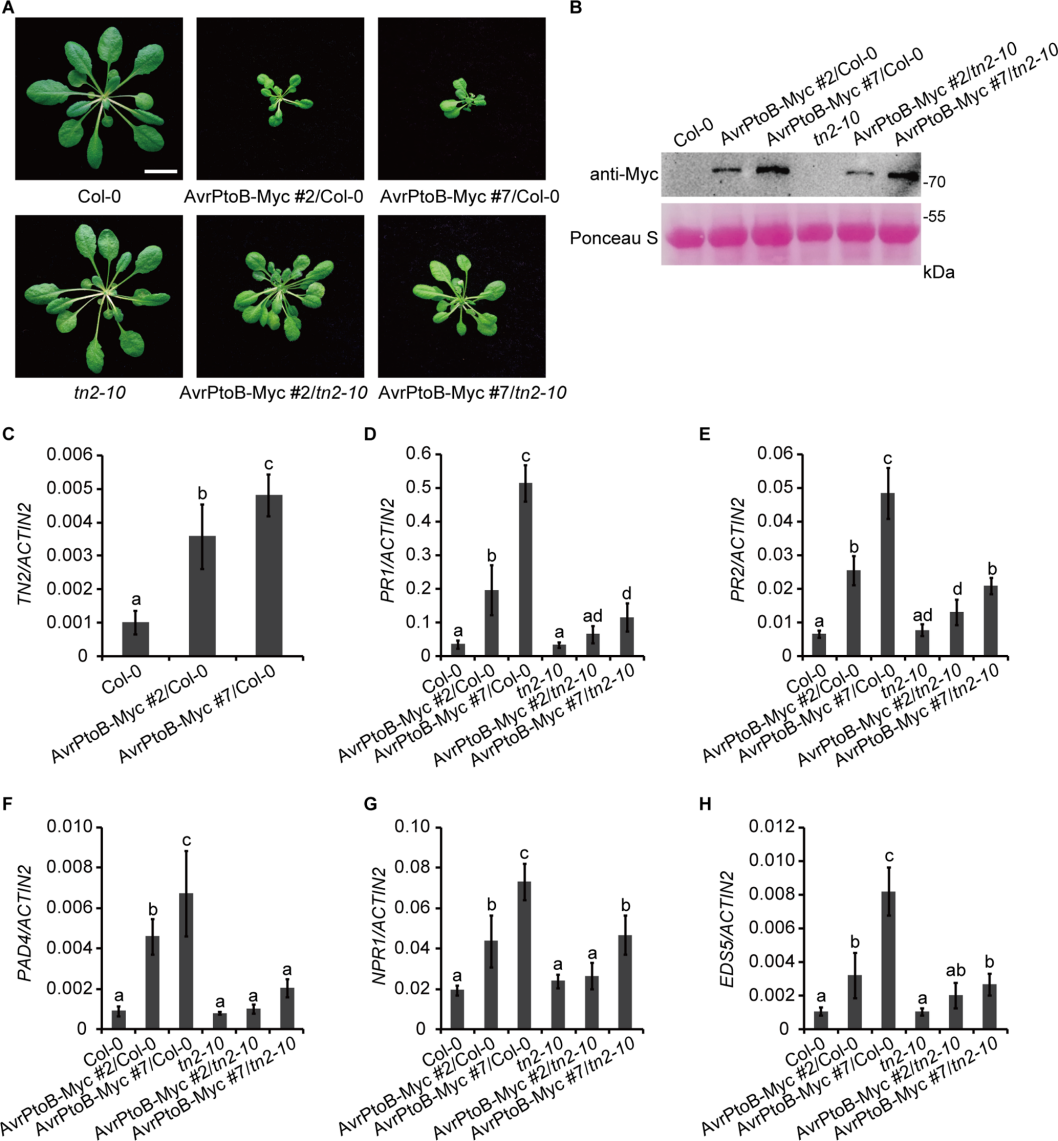
The stunted phenotypes of the AvrPtoB overexpression lines are partially dependent on TN2. **(A)** Five-week-old Col-0, *tn2-10*, and two independent *35S:AvrPtoB-Myc* transgenic *Arabidopsis* plants (*35S:AvrPtoB-Myc* #2 and *35S:AvrPtoB-Myc* #7) in the Col-0 and *tn2-10* background were photographed under short-day conditions. Bar = 1.0 cm. **(B)** The AvrPtoB-Myc fusion protein was examined by immunoblotting. Ponceau S staining of Rubisco was shown as a protein loading control. **(C–H)** RT-qPCR analysis of *TN2* **(C)**, *PR1* **(D)**, *PR2* **(E)**, *PAD4* **(F)**, *NPR1* **(G)**, and *EDS5* **(H)** expression. Total RNA was isolated from leaves of the indicated 5-week-old *Arabidopsis* plants. The expression levels of indicated gene were normalized to the reference gene *ACTIN2*. Data represent the mean and standard deviation of three biological replicates. Three technical replicates for each biological sample were used. The lowercase letters indicate statistically significant differences (*P* < 0.05; one-way ANOVA). These experiments were repeated three times with similar results.

To investigate whether there is a direct link between the phenotypes of *35S:AvrPtoB-Myc* plants and the accumulation of TN2, we crossed the *35S:AvrPtoB-Myc* #2 and #7 plants with the *tn2-10* mutant, a T-DNA insertion mutant of *TN2*. The *tn2-10* mutation fully suppressed the spontaneous cell death and powdery mildew resistance in the *exo70B1-3* mutant, indicating that *tn2-10* is a loss-of-function mutation (**Supplementary Figures S3A–C**). The expression level of *TN2* was up-regulated in the *exo70B1-3* mutants, which is consistent with our early reports (Zhao et al., 2015; Liu et al., 2017), and was not detected in *tn2-10* mutants and *exo70B1-3 tn2-10* double mutants (**Supplementary Figure S3D**). We obtained stable *35S:AvrPtoB-Myc* transgenic plants in the Col-0 (*35S:AvrPtoB-Myc* #2/Col-0 and *35S:AvrPtoB-Myc* #7/Col-0) and *tn2-10* mutant (*35S:AvrPtoB-Myc* #2/*tn2-10* and *35S:AvrPtoB-Myc* #7/*tn2-10*) background by crossing. We observed the stunted phenotypes of the *35S:AvrPtoB-Myc* #2 and #7 plants in the Col-0 background, which was indistinguishable from the phenotypes of the *35S:AvrPtoB-Myc* #2 and #7 plants in the background of the *exo70B1-3* transgenic line. In contrast, the stunted phenotypes were partially rescued by the *tn2-10* mutation (**Figure 5A**). The levels of AvrPtoB-Myc were similar in the *35S:AvrPtoB-Myc* #2 and #7 plants in the Col-0 and *tn2-10* background (**Figure 5B**), indicating that the stunted phenotype caused by the expression of AvrPtoB-Myc is partially dependent on TN2.

Consistent with the phenotypes, the transcript levels of *TN2* were up-regulated in *35S:AvrPtoB-Myc* #2/Col-0 and *35S:AvrPtoB-Myc* #7/Col-0 plants (**Figure 5C**), and the up-regulation of *PR1*, *PR2*, *PAD4*, *NPR1*, and *EDS5* expression levels was partially suppressed by the *tn2-10* mutation (**Figures 5D–H**). Taken together, these data indicated that overexpression of AvrPtoB in *Arabidopsis* causes a decrease of EXO70B1 and activates TN2-triggered immunity.

### AvrPtoB Overcomes EXO70B1-Mediated Resistance

**Figure 6:**
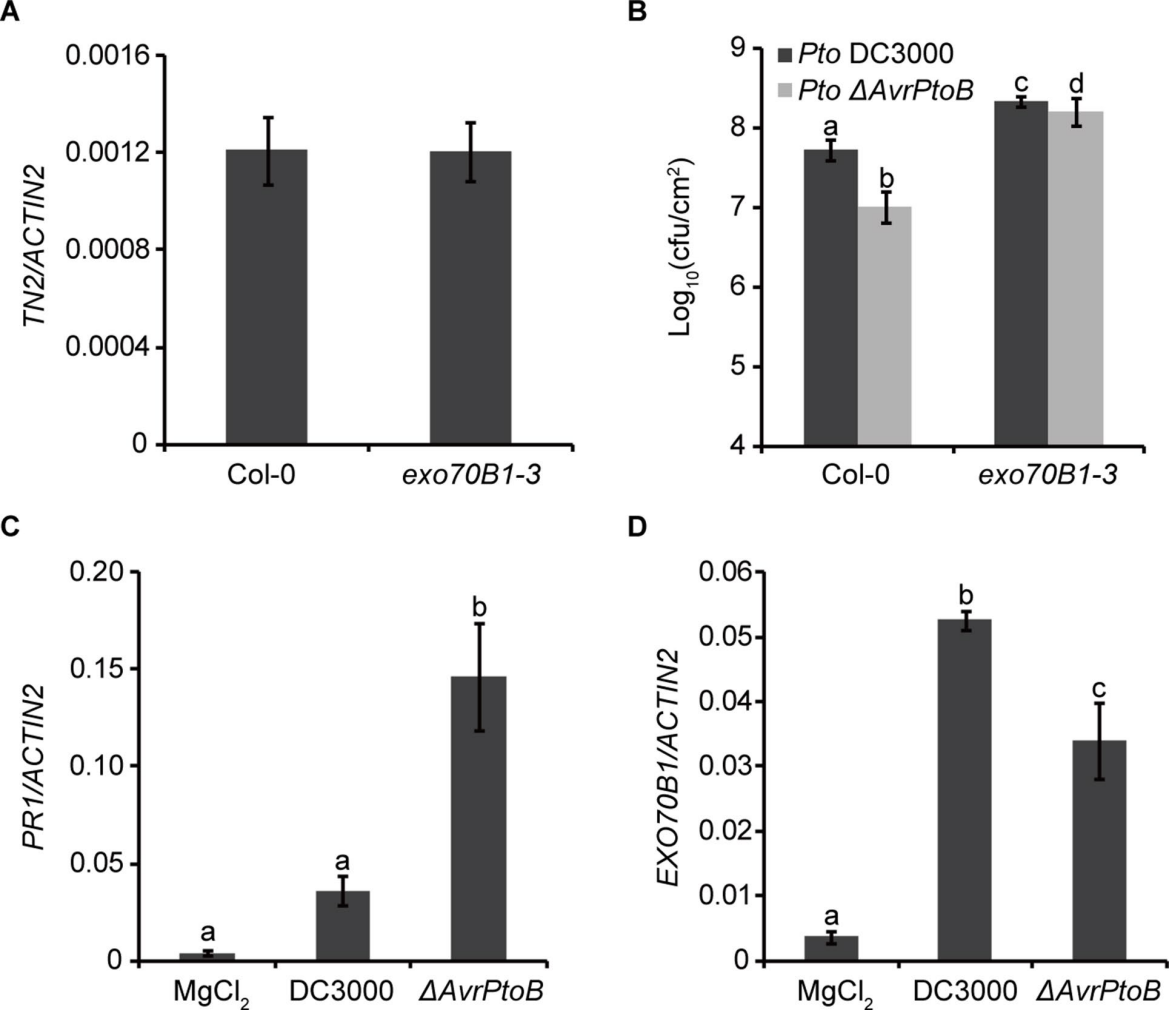
AvrPtoB overcomes EXO70B1-mediated resistance. **(A)** *TN2* transcript levels were examined by RT-qPCR. Total RNA was isolated from the indicated 10-day-old seedlings. The mRNA expression level was normalized to the reference gene *ACTIN2*. Data represent the mean and standard deviation of three biological replicates. Three technical replicates for each biological sample were used. This experiment was repeated three times with similar results. **(B)** AvrPtoB contributes to the virulence of *Pto* DC3000, which is dependent on its ability to disable the function of EXO70B1. Ten-day-old Col-0 and *exo70B1-3* seedlings were infected with *Pto* DC3000 and *Pto ΔAvrPtoB*. The bacterial growth assays were performed 3 days after infection. Results represent the mean and standard deviation of three independent experiments each consisting four independent biological samples. cfu, colony-forming units. The lowercase letters indicate statistically significant differences (*P* < 0.05; nested ANOVA). **(C)** and **(D)** *Pto* DC3000 enhances the expression of *EXO70B1*. Leaves of soil-grown wild-type Col-0 *Arabidopsis* plants were hand infiltrated with *Pto* DC3000 or the *Pto ΔAvrPtoB* mutant at 10^8^ cfu/ml and harvested at 24-h postinoculation. The transcript levels of *PR1* and *EXO70B1* were measured by RT-qPCR as described above. Data represent the mean and standard deviation of three biological replicates. Three technical replicates for each biological sample were used. These experiments were repeated three times with similar results. The lowercase letters indicate statistically significant differences (*P* < 0.05; one-way ANOVA).

Previously, we showed that *TN2* transcripts were up-regulated only in mature *exo70B1-3* plants (Zhao et al., 2015), and the expression level of *TN2* in 10-day-old *exo70B1-3* seedlings was similar to that in Col-0 (**Figure 6A**). However, why the *TN2* mRNA level did not change in young seedlings of *exo70B1-3* mutant is still unclear. As the *TN2* mRNA level did not change in young seedlings of *exo70B1-3* mutant, the phenotypes of young *exo70B1-3* seedlings would reflect the real role of EXO70B1 in resistance. Therefore, to assess whether degradation of EXO70B1 by AvrPtoB is important for the proliferation of *Pto* DC3000 in its host, we examined 10-day-old *exo70B1-3* seedlings for responses to *Pto* DC3000 and the *Pto* DC3000 mutant strain lacking *AvrPtoB* (*Pto ΔAvrPtoB*). The virulence of the *Pto ΔAvrPtoB* strain was strongly reduced in Col-0 (**Figure 6B**), which is consistent with earlier reports (He et al., 2006; Gohre et al., 2008). In the *exo70B1-3* mutant seedlings, bacterial numbers increased to similar extents for both *Pto* DC3000 strains, indicating that EXO70B1 contributes to bacterial resistance in *Arabidopsis*, and AvrPtoB enhances the virulence of *Pto* DC3000 by overcoming EXO70B1-mediated resistance.

To gain more insight into the function of EXO70B1 in plant immunity, we analyzed the transcripts of *EXO70B1* as well as *PR1* in Col-0 plants after inoculation with *Pto* DC3000 and *Pto ΔAvrPtoB*. Similar to the recent report, the *PR1* transcript level was lower in plants inoculated with *Pto* DC3000 than with *Pto ΔAvrPtoB* (Chen et al., 2017) (**Figure 6C**). The expression levels of *EXO70B1* were up-regulated after inoculation with both strains, and the up-regulation was enhanced in the presence of AvrPtoB (**Figure 6D**).

### EXO70B1 Suppresses TN2-Triggered Cell Death in Tobacco

**Figure 7:**
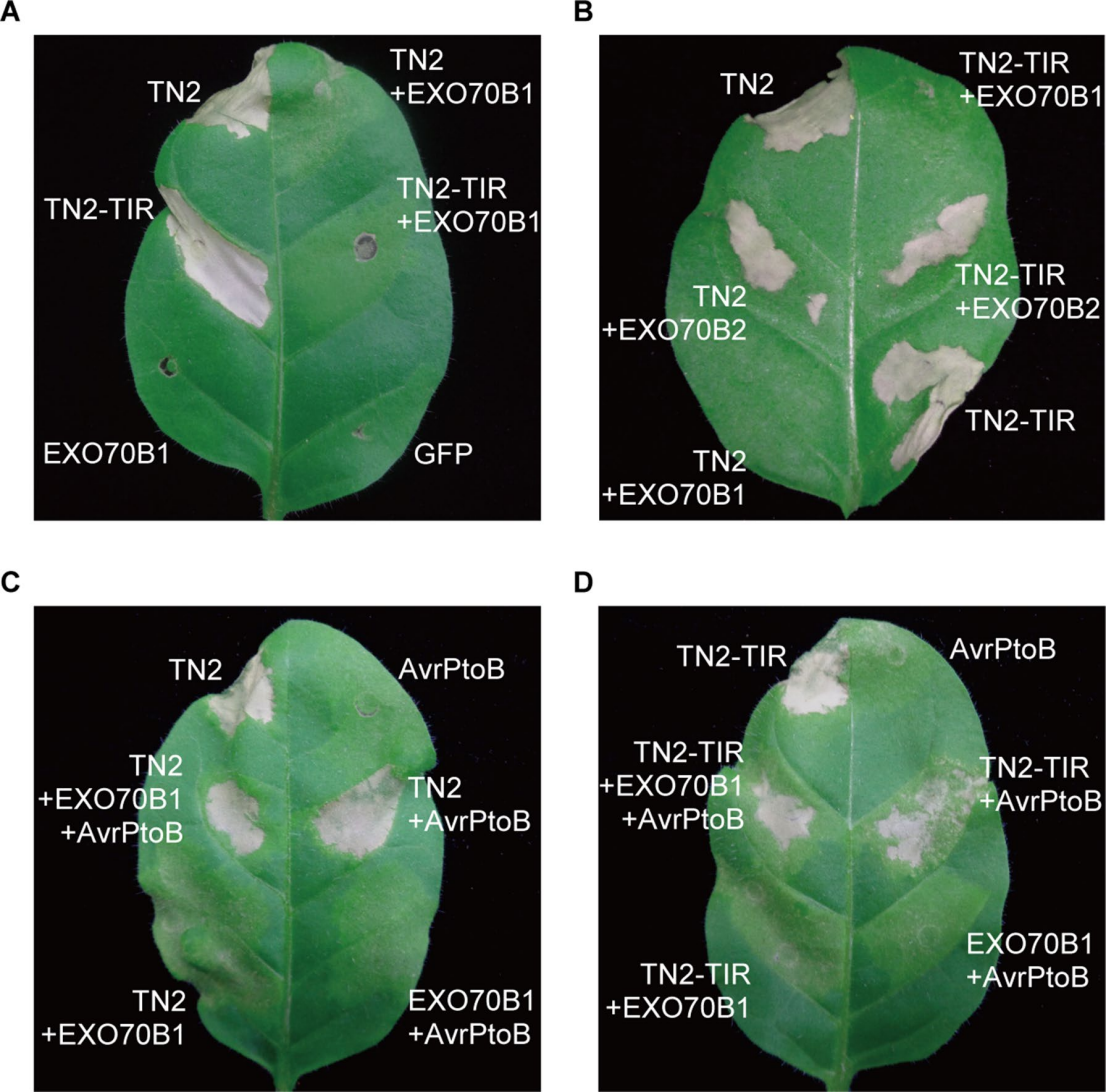
EXO70B1 suppresses TN2- or TN2-TIR-triggered cell death in *N. tabacum*. **(A)** and** (B)** TN2- or TN2-TIR-triggered cell death was suppressed by EXO70B1, but not EXO70B2, in *N. tabacum*. **(C)** and **(D)** AvrPtoB rescues TN2- or TN2-TIR-triggered cell death, which is suppressed by EXO70B1, in *N. tabacum*. *Agrobacterium* GV3101 cells carrying different constructs were mixed prior to adding infiltration buffer. The concentration of each agrobacteria in the mix was brought to OD_600_ = 0.5 and infiltrated into 5-week-old *N. tabacum* leaves for transient expression. Cell death was observed 36 to 48 h later. Pictures were taken 4 days postinfiltration. These experiments were repeated three times with similar results.

To further investigate the relationship between EXO70B1 and TN2, we expressed these proteins in *N. tabacum*. The expression of TN2 or TN2-TIR triggered a strong and rapid hypersensitive response (HR). This TN2- or TN2-TIR-triggered cell death was suppressed by co-expression with EXO70B1, not EXO70B2 (**Figures 7A, B**). TN2- or TN2-TIR-triggered cell death was also suppressed when we co-expressed TN2 or TN2-TIR with EXO70B1 using different *Agrobacterium* GV3101 concentrations, and proteins of the correct size were detected by immunoblotting analysis (**Supplementary Figure S4**). These results indicated that EXO70B1 may maintain TN2 in an inactive status, and TN2 triggers cell death only in the absence of EXO70B1.

To further study the role of AvrPtoB in TN2-triggered cell death, we co-expressed TN2 or TN2-TIR with EXO70B1 and AvrPtoB in *N. tabacum*. As shown in **Figures 7C, D**, AvrPtoB rescued TN2- or TN2-TIR-triggered cell death, which was suppressed by EXO70B1. Similar results were observed in *N. benthamiana* leaves (**Supplementary Figure S5**). Taken together, these data indicated that EXO70B1 associates with TN2 and suppresses the activation of TN2, and TN2 is activated after the degradation of EXO70B1 by AvrPtoB in both *N. tabacum* and *N. benthamiana*.

## Discussion

EXO70B1 is a component of the exocyst complex and belongs to the EXO70 protein family. In this study, we show that EXO70B1 is required for early immune signaling events, such as the ROS burst and callose deposition, triggered by flg22, elf18, and chitin (**Figure 1**), which is consistent with the previous report on the requirement for EXO70B1 in flg22-triggered ROS burst (Stegmann et al., 2012). We also show that AvrPtoB, an effector of *Pto* DC3000, targets EXO70B1 to suppress plant immunity (**Figures 2**, **3**, and **6**). Overexpression of AvrPtoB in *Arabidopsis* leads to autoimmunity, which is partially dependent of TN2 (**Figures 4** and **5**). In addition, EXO70B1 inhibits the cell death triggered by TN2 (**Figure 7** and **Supplementary Figure S4**), suggesting that EXO70B1 associates with TN2 and may maintain TN2 in an inactive status.

In vesicle trafficking, exocytosis plays fundamental roles by maintaining membrane integrity and contributing to membrane remodeling in response to altered environmental conditions (Ding et al., 2011). RIN4 recruits EXO70B1 to the PM, and AvrRpt2 can release both RIN4 and EXO70B1 to the cytoplasm, indicating that the localization of EXO70B1 at PM may be required for plant immunity (Sabol et al., 2017). The *exo70B1-3* mutant was compromised in early responses to multiple PAMPs, which is likely due to impaired PRRs signaling. Future studies will need to determine whether EXO70B1-associated exocyst complex contributes to the recycling of important components in PTI signaling. Despite the importance of the exocyst complex in vesicle trafficking and immune signaling, very few effectors are reported to target the components of the exocyst complex. In rice, the effector Avr-Pii from the rice blast fungus Magnaporthe oryzae interacts with OsEXO70F2 and OsEXO70F3, and OsEXO70F3, not EXO70F2, is specifically involved in Pii-dependent resistance (Fujisaki et al., 2015). Similarly, in this study, we show that both EXO70B1 and EXO70B2 interact with AvrPtoB (**Figure 2** and **Supplementary Figure S2**), but only EXO70B1 is ubiquitinated by AvrPtoB (**Figure 3** and **Supplementary Figure S2**). These results indicated that AvrPtoB associates with both EXO70B1 and EXO70B2 as EXO70B1 and EXO70B2 show the highest sequence similarity, but AvrPtoB specifically ubiquitinates and mediates the degradation of EXO70B1 to suppress EXO70B1-related plant immune responses.

The amino acid residues 206–307 of AvrPtoB, also known as the NPR1-interacting domain (NID) (Chen et al., 2017), are necessary for the interaction between AvrPtoB and EXO70B1 (**Figure 2D**). This domain is sufficient for the interaction between AvrPtoB and most of its targets, including NPR1, MKK2 (MAP kinase kinase 2), Bti9 (AvrPtoB tomato-interacting protein 9), and CERK1 (Gimenez-Ibanez et al., 2009; Zeng et al., 2012; Wei et al., 2015; Chen et al., 2017). Besides the NID, the Fen-interacting domain (FID) of AvrPtoB, encoded by amino acid residues 308–387, is another important region for the interaction between AvrPtoB and its targets, including Fen, FLS2, and BAK1 (Rosebrock et al., 2007; Gohre et al., 2008). Among the AvrPtoB-targeted proteins, only Pto binds to the Pto-interacting domain (PID) between amino acids 121 and 200, and to the FID domain of AvrPtoB, all other targets of AvrPtoB bind to the 206–387 amino acid region (NID and FID) of AvrPtoB. However, no interaction was observed between the full-length AvrPtoB and EXO70B1 in Y2H assay maybe because of the AvrPtoB E3 ligase activity. It is likely in the early report that the E3 ligase-deficient AvrPtoB mutants, not wild-type AvrPtoB, interacted with Fen in Y2H assay (Rosebrock et al., 2007). Further study is also needed to investigate why the N-terminal region of AvrPtoB can target so many different proteins, including protein kinases, transcriptional coactivator NPR1, and the subunit of exocyst complex EXO70B1.

AvrPtoB targets a number of regulators of plant immunity and contributes to the virulence of *Pto* DC3000. However, there is no evidence that any targets of AvrPtoB are monitored by NLR proteins and trigger ETI in *Arabidopsis* when targeted. Here, we showed that AvrPtoB ubiquitinates and degrades EXO70B1, and overexpression of AvrPtoB in *Arabidopsis* leads to autoimmunity, which is similar to the phenotype of TN2 overexpression plants reported previously (Li et al., 2009; Nandety et al., 2013), but not to the phenotype of *exo70B1* mutants (**Figure 4**). The most like reason is that the *TN2* transcripts were only up-regulated in older *exo70B1-3* plants (Zhao et al., 2015), which is different from *TN2* overexpression plants. Therefore, AvrPtoB overexpression phenotypes maybe due to the consistent up-regulation of TN2. Moreover, the autoimmunity phenotypes of the *35S:AvrPtoB-Myc* transgenic plants are partially dependent on TN2 (**Figure 5**). The reason why the *tn2-10* mutation does not fully rescue the stunted phenotypes of the *35S:AvrPtoB-Myc* transgenic plants may be because there are many targets of AvrPtoB in *Arabidopsis* and AvrPtoB may trigger other forms of NLR-mediated resistance. For instance, AvrPtoB also targets BAK1, and loss-of-function *bak1* mutants show a cell death phenotype (Kemmerling et al., 2007), which could be caused by activation of an undefined NLR protein.

*Pto* DC3000, a bacterial strain carrying AvrPtoB, did not show a reduced fitness postinoculation to Col-0 (**Figure 6B**). The most likely explanation for this is that AvrPtoB targets lots of proteins functioning in plant immunity, and modifications of other targets by AvrPtoB may contribute to its virulence, which overcome TN2-mediated resistance. For instance, NPR1, which contributes to TN2-mediated resistance (Zhao et al., 2015), is also targeted by AvrPtoB (Chen et al., 2017), and the modification of NPR1 by AvrPtoB could interfere with TN2-mediated resistance. In addition, the amount of AvrPtoB secreted into plant cells by *Pto* DC3000 is limited, which is insufficient to trigger TN2-mediated resistance. Furthermore, several other effectors can interact with EXO70B1, and those effectors may modify EXO70B1 as well. The modification of EXO70B1 by other effectors may influence the ubiquitination and degradation of EXO70B1 mediated by AvrPtoB. Therefore, TN2-mediated resistance is not activated.

Ectopic expression of the full-length or truncated TIR domain of some NLR proteins in the absence of pathogens can trigger an HR in *N. tabacum* (Swiderski et al., 2009; Bernoux et al., 2011). In this study, we show that transient expression of TN2 or TN2-TIR alone triggers strong and rapid cell death in *N. tabacum*, which is suppressed by co-expression of EXO70B1, not EXO70B2 (**Figure 7**), indicating that EXO70B1 specifically suppresses the activation of TN2, and which is consistent with results on TN2 interaction with EXO70B1, but not with EXO70B2 (**Supplementary Figure S2A**). Additionally, TN2 and TN2-TIR accumulated in the presence of EXO70B1 (**Supplementary Figure S4**), indicating that EXO70B1 associates with TN2 and may maintain TN2 in an inactive status, rather than to degrade TN2 by EXO70B1-associated autophagy activity.

The genome of the *Arabidopsis* ecotype Col-0 encodes about 50 atypical NLR proteins, including TIR, TIR-X, and TIR-NBS (Meyers et al., 2002), some of which have been reported to play roles in plant immunity. For instance, RESPONSE TO HOPBA1 (RBA1), a TIR-only protein, functions as a pathogen sensor and recognizes the HopBA1 effector directly (Nishimura et al., 2017). TN13 is a MODIFIER OF SNC1, 6 (MOS6) interaction partner required for resistance against *Pto* DC3000 lacking AvrPto and AvrPtoB (Roth et al., 2017). However, how truncated NLR proteins activate disease resistance is not clear. It is proposed that the truncated NLR proteins might not function alone and instead play a role in immunity with full-length NLR proteins (Nandety et al., 2013). Future studies are needed to determine whether overexpression of AvrPtoB triggered autoimmunity and TN2-activated resistance rely on full-length NLR proteins.

## Conclusion

Our study demonstrated that EXO70B1, a subunit of the exocyst complex, plays an important role in immune responses triggered by multiple PAMPs. *Pto* DC3000 secretes AvrPtoB into plant cells to target EXO70B1, which may contribute to the trafficking of plasma membrane proteins involved in PAMP-triggered immunity. EXO70B1 associates with and maintains TN2, a truncated NLR protein, in an inactive status. Although the role of EXO70B1 in plant immunity and how EXO70B1 inhibits TN2 activation need further study, our results highlight that the *Arabidopsis* exocyst component is a target of effectors and may be guarded by a truncated NLR protein.

## Data Availability

Sequence data from this article can be found in the Arabidopsis Genome Initiative or GenBank/EMBL databases under the following accession numbers: AT5G03540 (At EXO70A1), At5g58430 (At EXO70B1), At1g07000 (At EXO70B2), At1g17615 (At TN2), At2g14610 (At PR1), AT3g57260 (At PR2), At3g52430 (At PAD4), At1g64280 (At NPR1), At4g39030 (At EDS5), At3g18780 (At ACTIN2), AAF76306 (Sl Pto), AAO57459 (AvrPto), AY074795 (AvrPtoB), AAO54130 (Hop H1), AAO53599 (Hop K1), AF458392 (Hop O1-1), and AF458403 (Hop Y1).

## Author Contributions

DT and WW conceived and initiated the research and designed the experiments; WW, NL, CG, and LR performed the experiments; WW, NL, CG, and DT analyzed the data; DT and WW wrote the paper.

## Funding

The work was supported by grants from the National Natural Science Foundation of China (31761133017) and National Science Fund for Distinguished Young Scholars of China (31525019) to DT.

## Conflict of Interest Statement

The authors declare that the research was conducted in the absence of any commercial or financial relationships that could be construed as a potential conflict of interest.
