# Supplementary material for: The Pseudomonas Syringae Effector AvrPtoB Associates With and Ubiquitinates Arabidopsis Exocyst Subunit EXO70B1
Source: Front Plant Sci. 2019 Aug 29;10:1027. doi: 10.3389/fpls.2019.01027 (PMC6726739; doi:10.3389/fpls.2019.01027)
Supplement: Supplementary file 1 [file DataSheet_1.pdf]

Supplementary Figures

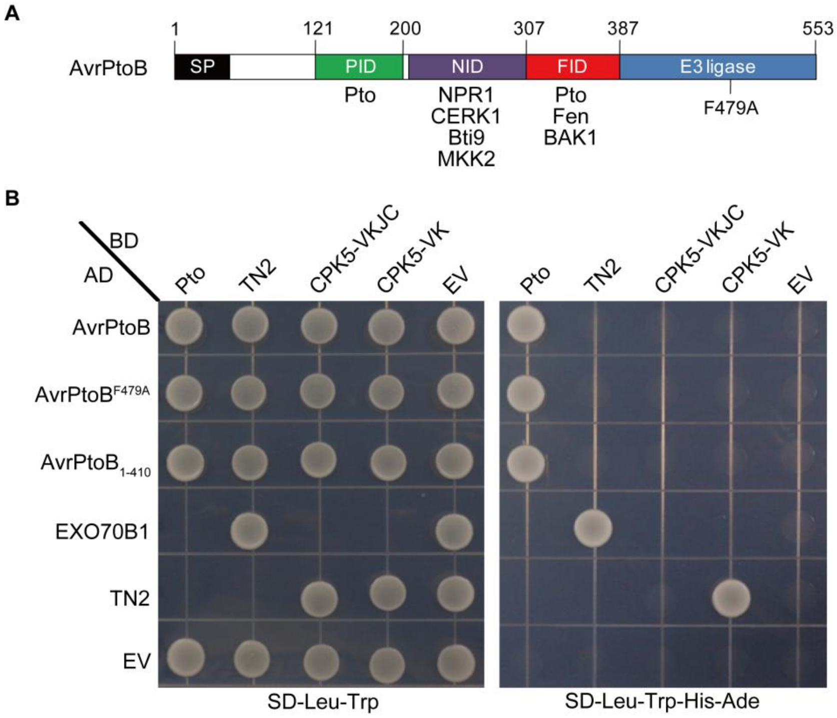

Figure S1. No interaction was observed between AvrPtoB and TN2 or CPK5 in Y2H assays. (A) A schematic diagram of the AvrPtoB. Each box indicates the specific domain of AvrPtoB, including the predicted signal peptide (SP), Pto-interacting domain (PID), NPR1-interacting domain (NID), Fen-interacting domain (FID) and E3 ubiquitin ligase region. F479A indicated the point mutation of AvrPtoB in E3 ligase domain. **(B)** AvrPtoB, AvrPtoB^{F479A}, and AvrPtoB$_{1\text{-}410}$ were fused to the AD, and TN2, CPK5-VKJC and CPK5-VK were fused to the BD. Different pairs of constructs were cotransformed into AH109. Yeast cells containing the indicated plasmids were spotted onto SD-Leu-Trp or SD-Ade-His-Leu-Trp media. Photographs were taken 5 days after incubation. No yeast cells were dropped on the blank areas of SD-Leu-Trp media and these same areas on SD-Ade-His-Leu-Trp media. This experiment was repeated three times with similar results.

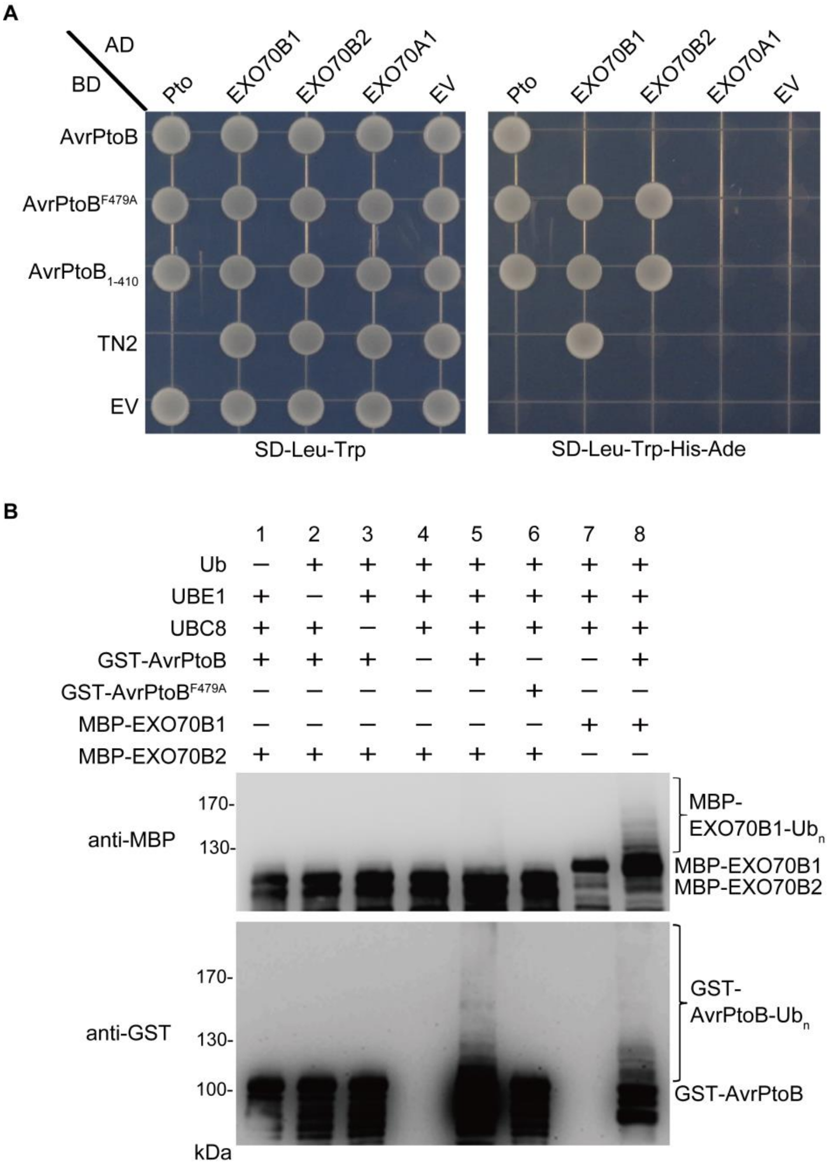

Figure S2. EXO70B2 is not a substrate of AvrPtoB. (A) EXO70B1, EXO70B2 and EXO70A1 were fused to the AD, and AvrPtoB, AvrPtoB^{F479A}, and AvrPtoB$_{1-410}$ were fused to the BD. Different pairs of constructs were cotransformed into AH109. Yeast cells containing the indicated plasmids were spotted onto SD-Leu-Trp or SD-Ade-His-Leu-Trp media. Photographs were taken 5 days after incubation. No yeast cells were dropped on the blank areas of SD-Leu-Trp media and these same areas on SD-Ade-His-Leu-Trp media. **(B)** *In vitro* ubiquitination assay. Recombinant MBP-EXO70B2 incubated in ubiquitination buffer in the presence or absence of ubiquitin, E1 (UBE1), E2 (UBC8) or E3 (AvrPtoB or AvrPtoB^{F479A}). MBP-EXO70B1 was used for a positive control. The reaction mixtures were subjected to immunoblot analysis with anti-MBP and anti-GST antibodies. Ubiquitinated MBP-EXO70B1 was detected by anti-MBP antibody. These experiments were repeated three times with similar results.

.

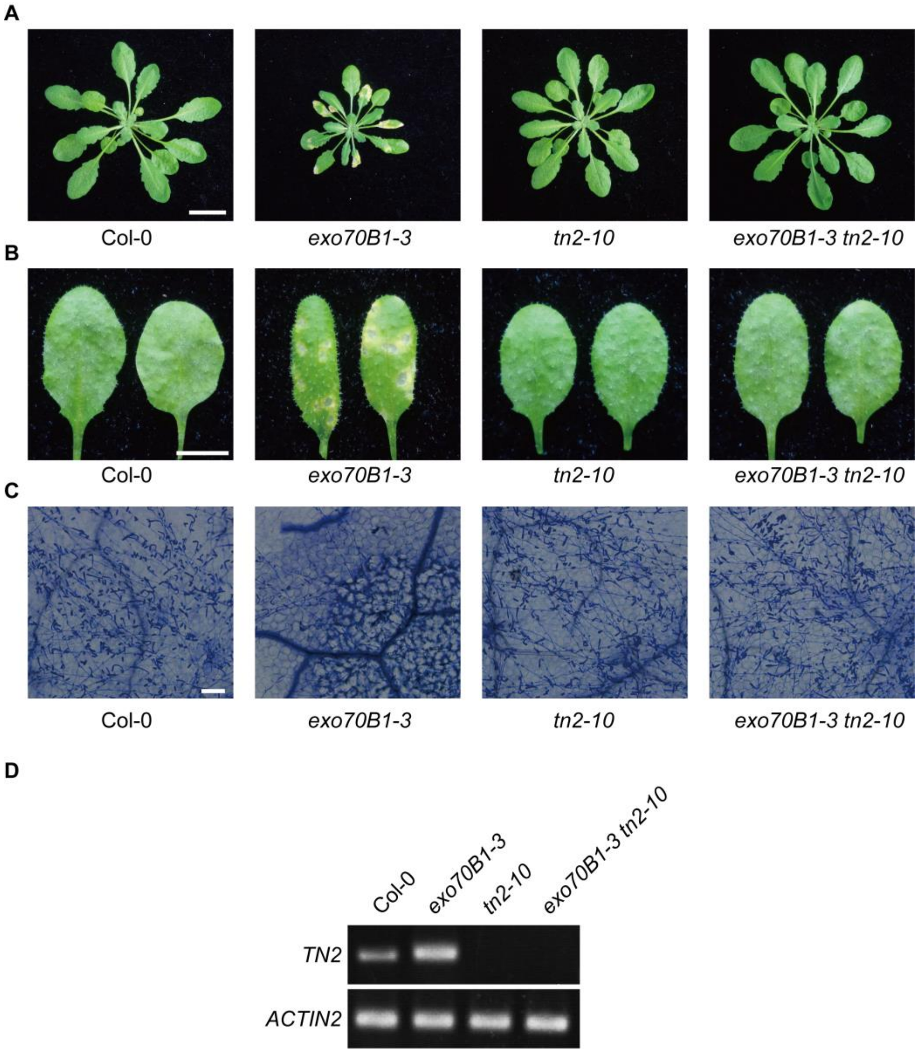

Figure S3. The *tn2-10* mutation suppresses *exo70B1-3*-mediated cell death and resistance to *G. cichoracearum*. (A) Five-week-old plants were photographed under short-day conditions. The *exo70B1-3* mutants displayed hypersensitive responses, but no cell death was observed in Col-0, *tn2-10* and *exo70B1-3 tn2-10* mutants. Bar = 1.0 cm. **(B)** Four-week-old plants were infected with *G. cichoracearum*. Representative leaves were detached and photographed at 8 days post infection. The *exo70B1-3* mutant was more resistant to *G. cichoracearum*, while the *tn2-10* and *exo70B1-3 tn2-10* mutants showed wild-type like responses to *G. cichoracearum*. Bar = 0.5 cm. **(C)** Leaves at 8 days post infection were stained with trypan blue to observe fungal structures and dead cells. Few spores were produced in the *exo70B1-3* mutant, whereas many fungal spores were produced in Col-0, *tn2-10*, and *exo70B1-3 tn2-10* plants. Bar = 100 μm. **(D)** The transcript level of *TN2* was examined by RT-qPCR. Total RNA was isolated from the indicated five-week-old plants shown in **(A)**. The transcripts of *TN2* were up-regulated in *exo70B1-3* mutants and were not detected in *tn2-10* and *exo70B1-3 tn2-10* plants. These experiments were repeated three times with similar results.

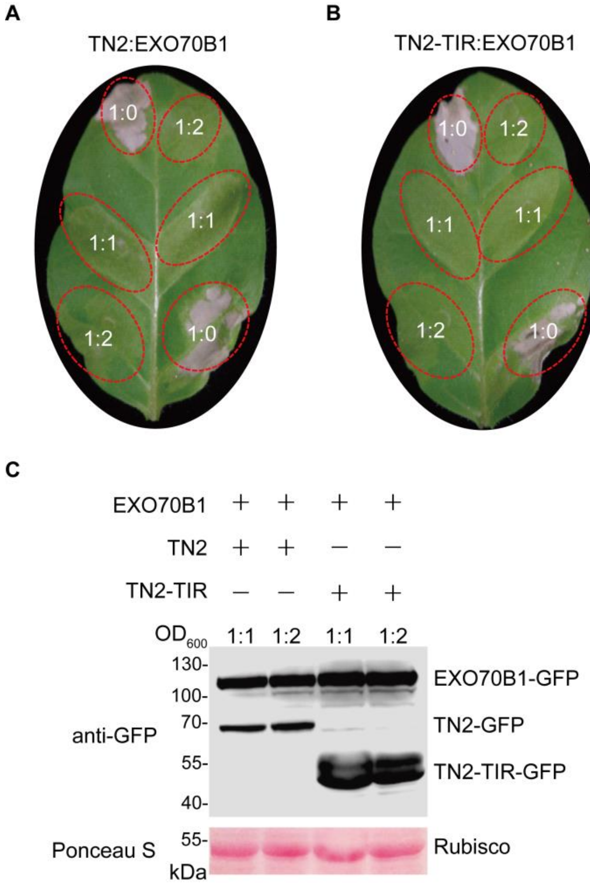

Figure S4. EXO70B1 suppresses TN2- or TN2-TIR-triggered cell death in *N. tabacum*. (A) and **(B)** TN2- or TN2-TIR-triggered cell death was suppressed by EXO70B1 in *N. tabacum*. *Agrobacterium* GV3101 cells carrying different constructs were mixed prior to adding infiltration buffer. The concentration of agrobacteria expressing *TN2* or *TN2-TIR* was brought to OD_{600} = 0.5 and the concentration of agrobacteria expressing *EXO70B1* was brought to OD_{600} = 0, 0.5, or 1.0. Five-week-old *N. tabacum* leaves were used for transient expression. Cell death was observed 36 to 48 h later. Pictures were taken 4 days post infiltration. Dashed lines mark the infiltrated areas. **(C)** Accumulation of the indicated recombinant proteins from the experiments in **(A)**, which failed to trigger a HR. Proteins were detected by anti-GFP antibody and equal loading is shown by Rubisco. These experiments were repeated three times with similar results.

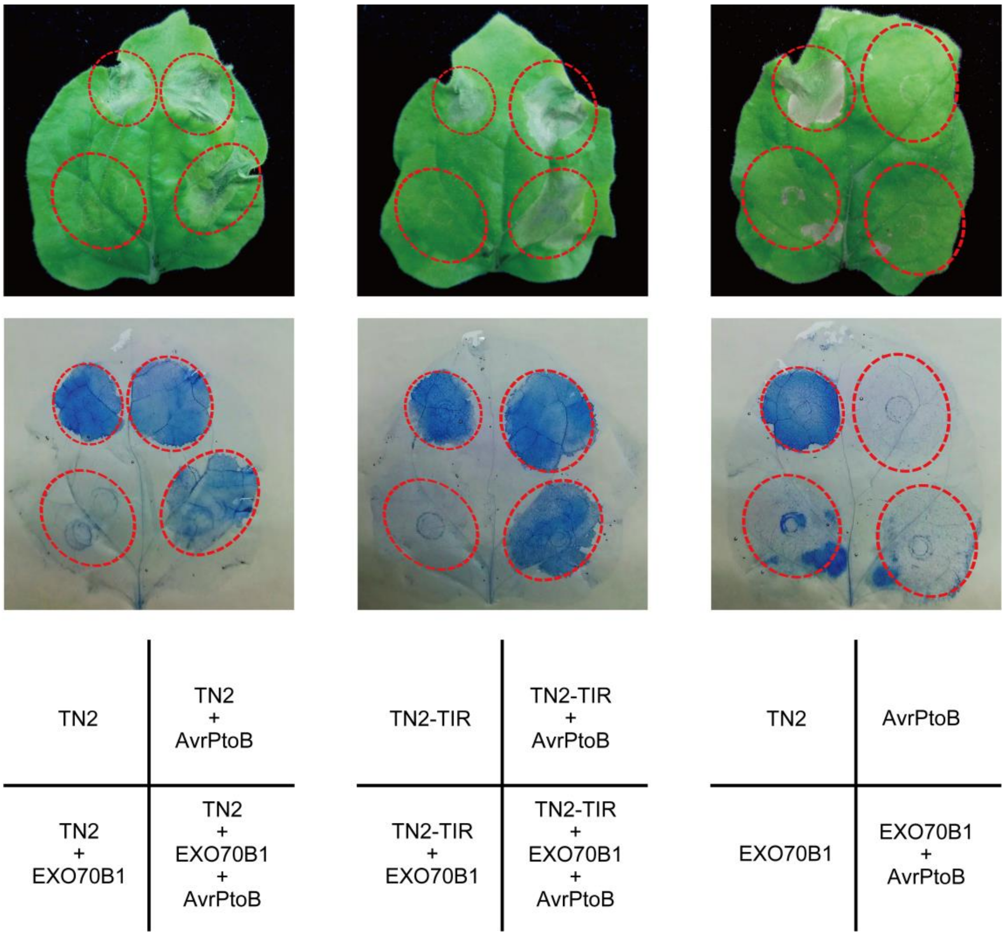

Figure S5. AvrPtoB rescues TN2- or TN2-TIR-triggered cell death, which is suppressed by EXO70B1, in *N. benthamiana*. *Agrobacterium* GV3101 cells carrying different constructs were infiltrated into five-week-old *N. benthamiana* leaves. Pictures were taken 4 days post infiltration (upper panel). Leaves at 4 days post infiltration were stained with trypan blue to visualize cell death (lower panel). Dashed lines mark the infiltrated areas. These experiments were repeated three times with similar results.

Supplementary Table 1. Primers used in this study.

Primer name	Purpose	Sequence (5'-3')
AvrPto PF	Yeast two-hybrid	GGAATTCCATATGATGGGAAATATATGTGTCGG
AvrPto PR	Yeast two-hybrid	CGGAATTCTCATTGCCAGTTACGGTACGG
AvrPtoB PF	Yeast two-hybrid	GGAATTCCATATGATGGCGGGTATCAATAGAGC
AvrPtoB PR	Yeast two-hybrid	CGGAATTCTCAGGGGACTATTCTAAAAGCATAC
AvrPtoB 1-410 PR	Yeast two-hybrid	CGGAATTCTCACCCCGGGTTCAGGTTAAG
AvrPtoB 1-205 PR	Yeast two-hybrid	CGGGATCCTCATGACGCCGCCTGTTGGT
AvrPtoB 1-307 PR	Yeast two-hybrid	CGGGATCCTCATACATGTCTTTCAAGGGCC
AvrPtoB 1-387 PR	Yeast two-hybrid	CGGGATCCTCACACCCGCAATCGTGTTG
AvrPtoB 308-533 PF	Yeast two-hybrid	GGAATTCCATATGATGCAGCGCCTCCCTATC
AvrPtoB 308-533 PR	Yeast two-hybrid	CGGGATCCTCAGGGGACTATTCTAAAAGCATAC
Pto PF	Yeast two-hybrid	GGAATTCCATATGATGGGAAGCAAGTATTCTAAGG
Pto PR	Yeast two-hybrid	CGGAATTCTTAAATAACAGACTCTTGGAGACGAA
EXO70A1 PF	Yeast two-hybrid	CGGAATTCATGGCTGTTGATAGCAGAATG
EXO70A1 PR	Yeast two-hybrid	AACTGCAGTTACCGGCGTGGTTCATT
EXO70B2 PF	Yeast two-hybrid	CGGAATTCATGGCTGAAGCCGGTGAC
EXO70B2 PR	Yeast two-hybrid	AACTGCAGTCAACTTGAGCTTTCCTTGA
avrPtoB M PF	Site-directed mutagenesis	TGACGCACTGTCTTGCTGGCGGAGAATTGT
avrPtoB M PR	Site-directed mutagenesis	ACAATTCTCCGCCAGCAAGACAGTGCGTCA
AvrPtoB PF	To clone into pGEX-4T-1	CGGAATTCATGGCGGGTATCAATAGAGC

AvrPtoB PR	To clone into pGEX-4T-1	TTGCGGCCGCGGGACTATTCTAAAAGCATAC
AvrPtoB 1-410 PR	To clone into pGEX-4T-1	TTGCGGCCGCTCACCCCGGGTTCAGGTTAAG
EXO70B1 PF	To clone into PUC19	CGAGCTCATGGCGGAGAATGGTGAA
EXO70B1 PR	To clone into PUC19	GCGTCGACTTTTCTTCCCGTGGTAG
AvrPtoB PF	To clone into pSuper1300-MYC	GCGTCGACATGGCGGGTATCAATAGAGC
AvrPtoB PR	To clone into pSuper1300-MYC	GGGGTACCGGGGACTATTCTAAAAGCATAC
AvrPtoB 1-410	To clone into pSuper1300-MYC	GGGGTACCCCCCGGGTTCAGGTTAAG
EXO70B1 PF	To clone into pSuper1300-GFP	GCGTCGACATGGCGGAGAATGGTGAA
EXO70B1 PR	To clone into pSuper1300-GFP	GGGGTACCTTTTCTTCCCGTGGTAG
LBb1.3	Genotyping	ATTTTGCCGATTTCGGAAC
TN2 Salk PF	Genotyping	TTCCAACAAAATCACCAGCTC
TN2 Salk PR	Genotyping	AAATCCCATCTGGGATTTGTC
TN2 PF	Real-time PCR	GGCTCATGAGTCAGAAAG
TN2 PR	Real-time PCR	GAAGATTCAGTCCCGGAT
PR1 PF	Real-time PCR	TTCACAACCAGGCACGAGGAG
PR1 PR	Real-time PCR	CTAACCCACATGTTCACGGCG
PR2 PF	Real-time PCR	GAATCAAGGAGCTTAGCCTCACC
PR2 PR	Real-time PCR	GTAGAGCCGCATTCGCTGGAT
PAD4 PF	Real-time PCR	CTTTCTTCAGTTAAAGATCAAGGAAGG
PAD4 PR	Real-time PCR	GGCAGAAGTTGTGTGCTAAACG
NPR1 PF	Real-time PCR	TAGAGTTGCACTTGCTCAACGTC
NPR1 PR	Real-time PCR	GTTTCCCGAGTTCCACGGT
EDS5 PF	Real-time PCR	GAACTCGCTGCTCTTGGACC
EDS5 PR	Real-time PCR	CAGCCCAAGGACCGAATAATC
ACTIN2 PF	Real-time PCR	TCTCCCGCTATGTATGTCGCC
ACTIN2 PR	Real-time PCR	GTCACGTCCAGCAAGGTCAAGA
EXO70B1 PF	Real-time PCR	GAAACCTATCGGCATCTGTC
EXO70B1 PR	Real-time PCR	CCGAAGGGAGAGCATCAAT
